# Post-marketing surveillance of anti-malarial medicines used in Malawi

**DOI:** 10.1186/s12936-015-0637-z

**Published:** 2015-03-25

**Authors:** Ibrahim Chikowe, Dorcas Osei-Safo, Jerry JEK Harrison, Daniel Y Konadu, Ivan Addae-Mensah

**Affiliations:** Department of Chemistry, University of Ghana, Legon, Accra, Ghana; Ministry of Education, Science and Technology, Lilongwe, Malawi

**Keywords:** Malawi, Anti-malarial medicines, Substandard, API, Post-marketing surveillance

## Abstract

**Background:**

The growing concern over the extent of anti-malarial medicine resistance in sub-Saharan Africa, driven largely by administration of sub-therapeutic doses derived from falsified and substandard medicines necessitates regular monitoring of the quality of these medicines to avert any potential public health disaster. This study aimed at determining the active pharmaceutical ingredient (API) content of anti-malarial medicines available in Malawi with respect to the manufacturers’ label claim and pharmacopoeia specifications.

**Methods:**

Samples of anti-malarial medicines (112) collected from both licensed and unlicensed markets throughout Malawi were subjected to visual inspection of dosage form and packaging, and registration verification with the regulatory body. Basic (colourimetric) tests were employed to establish the presence and identity of the requisite APIs. Semi-quantitative thin layer chromatography (SQ-TLC) was employed as a quick assay for the verification of identity and estimation of the API content while HPLC assays were used to quantify the APIs. The results were compared with pharmacopoeia specifications and manufacturers’ label claims. For combination therapies, a sample was considered to have failed if one or more of its component APIs did not meet pharmacopoeia specifications.

**Results:**

There was 86.6% registration status and 100% compliance with visual inspection and basic tests confirming the presence of requisite APIs. The identification test was confirmed by the SQ-TLC assay. API quantification by HPLC assay however, showed that 88.4% (99/112) of the samples failed the quality tests due to the presence of either insufficient or excessive API.

**Conclusions:**

The results suggest the existence of substandard anti-malarial medicines in Malawi. The presence of both excessive and insufficient artemisinin-based and non-artemisinin-based API, clearly points to poor adherence to GMP and improper handling during storage or distribution. The country relies heavily on imported anti-malarial medicines so there is an urgent need to carry out regular and thorough post-market surveillance of medicines to ensure better quality health care delivery.

**Electronic supplementary material:**

The online version of this article (doi:10.1186/s12936-015-0637-z) contains supplementary material, which is available to authorized users.

## Background

No other disease has killed more humans than malaria [1] and it is still claiming millions of lives worldwide. Approximately 3.2 billion people - about half of the world’s population - are at risk of malaria. In 2013, about 198 million cases were reported worldwide with an estimated 584,000 deaths. The WHO African Region recorded 90% of these deaths, mostly among children under five years of age [2]. Although, preventive and control measures introduced since 2000 are yielding positive results, leading to the reduction of mortality rates by 47% globally and 54% in Africa, the disease continues to be a major public health problem in most malaria-endemic countries [2]. As in most of sub-Saharan Africa [2], one hundred percent of the population in Malawi lives in a region of high malaria transmission. There is an estimated five million cases annually; responsible for about 30% of the outpatients treated at health facilities and about 40% of all hospitalizations of children under five years of age [3].

Malaria control strategic plans in Malawi comprise the following four key interventions: prompt access to ACT, intermittent preventive treatment during pregnancy (IPTP), long-lasting insecticide-treated nets (LLITNs), and indoor spraying with residual insecticides [3]. Since 2007, artemether-lumefantrine has been adopted as the first-line treatment for uncomplicated and unconfirmed cases, after replacing sulphadoxine-pyrimethamine (SP), which also replaced chloroquine in 1993 due to parasite resistance. SP and other anti-malarial medicines, such as quinine (QN), are still being used for special cases [2].

Plasmodium resistance to malaria treatment has several devastating effects. It has led to an increase in morbidity and mortality rate, parasite transmission, severity of the pandemic and change in malaria distribution. As a result, this has caused pressure on the economy due to increase in cost of health services arising from prevalent treatment failures and deaths [4]. Thus, patients resort to illegal medicines, exposing them to poor quality medicines. Fake/spurious/substandard/degraded/counterfeit medicines are mostly blamed for the escalation of medicine resistance. For example, some pockets of parasite resistance to artemisinin-based medicines have been attributed to the sub-therapeutic doses derived from falsified and substandard medicines [5]. Various studies have reported the widespread circulation of poor quality medicines in some parts of Asia and Africa. Most of them have been shown to contain sub-therapeutic amounts of the APIs or no API at all or even toxic compounds [6-8].

Currently, the only hope for future malaria treatment rests on artemisinin-based combination therapy (ACT). However, with the high demand and cost of production of these medicines, the poor regulatory systems that exist in most endemic countries including Malawi allow unscrupulous persons to easily infiltrate the weak chain supply systems with both imported and/or domestic poor quality medicines [9,10]. Therefore, it is imperative to protect these medicines from any impending medicine resistance through GMP, relentless combat against the circulation of poor quality medicines and strict patient compliance to treatment regimen.

The WHO Expert Committee on Quality Assurance of Medicines calls for routine quality control activities to check this malpractice. Therefore, this study aimed at evaluating the quality of the anti-malarial medicines used in Malawi with respect to the active pharmaceutical ingredient (API) content in both ACT and non-ACT. To achieve this aim, the following specific objectives were set: to find out the registration status of the anti-malarial medicines available on the markets; to visually inspect dosage forms and packaging using the guidelines outlined in the WHO pharmacopoeia and the literature; to carry out a qualitative determination to establish the presence or otherwise of the APIs using the authenticated rapid tests outlined in the WHO publications; to carry out a quantitative determination of the API content.

## Methods

### Sampling procedures

Samples were collected by the first author after seeking permission from the regulatory boards; the Pharmacy, Medicines and Poisons Board and the National Health Sciences Research Committee (NHSRC) of Malawi. The medicines were bought under the guise of a patient, but in the case where pharmacy technicians refused to sell without prescription or many brands of medicine were being bought at once, it was explained that they were for research purposes. In the situation where the investigator introduced himself as a researcher, he ensured that he was sold the medicines from the open shelves to avoid a tendency where vendors give out only authentic goods to regulatory authorities or researchers.

The country was divided into four zones based on the National Malaria Control Programme (NMCP) strategy partitions designated as south west (1), south east (2), central (3) and north (4) zones. Few districts from each zone were selected based on malaria prevalence rates, economic activities and geographical position (border towns). A master list of pharmacies and private health facilities in the districts of interest was compiled. The pharmacies were considered based on proportionate sampling, called probability proportionality to size (PPS), i.e. more samples from districts with more pharmacies/health facilities. The districts of Lilongwe, Mzuzu and Blantyre were further divided into Enumeration areas (EAs) as these had pharmacies in separate townships as well, unlike the other districts that had few pharmacies/private health facilities all clustered at one place. Each township acted as an EA. The simple random sampling (SRS) procedures were used to select the EAs because the EAs were few per district. The random walk method was used in the selection of pharmacies/health facilities from the EAs while for the rest of the districts all pharmacies were selected as there were few pharmacies. Samples were collected between December and January (within the rainy season of November to April) when Malawi records peak malarial transmission due to abundance of stagnant water points, which are favourable breeding grounds for the mosquito vector. The anti-malarial medicines were purchased from both licensed and unlicensed markets such as private pharmacies and hospitals, street vendors and shops. The samples were purchased regardless of size, company name, brand, product name, dosage form and strength, though not more than one sample of the same name, batch number and characteristics were bought at one outlet. They were labelled, recorded and kept in containers that protected them from extreme light, moisture, crushing, heat and mechanical shock. For more details of the samples see Additional file 1. The sampling sites are shown in Additional file 2.

### Reference standards

The Reference standards were purchased from the European Directorate for the Quality of Medicines and Healthcare (EDQM), France.

### Medicine analysis

#### Registration verification and visual inspection

The samples were subjected to registration verification with the medicine regulatory authority - the Pharmacy, Medicines and Poisons Board of Malawi after the collection exercise. This was followed by visual inspection with respect to technical regulatory information as outlined in the WHO International Pharmacopoeia [11,12].

### Basic/Colourimetric tests

To determine if the samples contained the APIs claimed by the manufacturers on the packaging materials, all of them were subjected to colourimetric tests using the suitable reactions and reagents outlined in the pharmacopoeias and the literature [13-15].

### Semi-quantitative thin layer chromatography (SQ-TLC) assay

SQ-TLC was employed as a quick assay for the verification of identity and estimation of the API content in the medicine samples according to published protocols [16,17].

### HPLC assay

HPLC procedures suitable for determining the API in each anti-malarial sample adopted from the pharmacopoeias and the literature [17-21] were employed. Calibration curves were prepared using varying concentrations of the various Reference Standards (RS). The Area Under the Curve (AUC) for each concentration was determined from six replicates and an average AUC was obtained. This data was used to generate calibration curves of a plot of average AUC against concentration (C) using Microsoft Excel and the slope of the graph, intercept, correlation coefficient (r^2^) as well as equation of the straight line, AUC = mC + b were deduced and calculated. The quantities of the APIs in the medicine samples then were calculated from their corresponding calibration curves. Six replicates were carried out for each API component and the mean and standard deviations were calculated (see Additional files 3 and 4).

#### Assay for artesunate in ATS/SP and ATS/SmP samples

The assay for ATS was adopted from Ranher *et al.* [18] with a few modifications as follows: column measurements: Discovery C-18 bonded, 5 μm, 25 cm x 4 mm; mobile phase: 70: 30 v/v, 1% triethylamine (TEA) in methanol: buffer (10 mM KH_2_PO_4_/ 85% H_3_PO_4_, pH = 2.5); retention time (average): 5.1 minutes; detection wavelength: 216 nm*;* flow rate: 1.2 mL/min.; volume of injection: 20 μL. For each ATS-containing sample, a quantity of the powdered dosage form equivalent to 10 mg of artesunate was weighed into a clean dry 10 mL volumetric flask. 5 mL of the mobile phase was then added and the mixture shaken for 15 minutes on an ultrasonic sonicator. Then, more mobile phase was added to the mark and the solution filtered through a 0.45 μm filter.

#### Assay for artemether and lumefantrine in ATM/LUM samples

The assay protocol for ATM and LUM was derived from modifications of a method developed by Arun and Smith [19]. The adapted method is as follows: column measurements: Hyperprep PEP 300A C4, 25 cm x 4.6 mm, 8 μm; mobile phase: 70: 30 v/v acetonitrile: 10 mM buffer consisting of KH_2_PO_4_ mixed with 1 mL of triethylamine per liter and pH changed to 2.5 using 85% H_3_PO_4_ mixture; retention time (average): ATM 2.5 minutes, LUM 3.0 minutes; detection wavelength: 216 nm*;* flow rate: 1.5 mL/min.; volume of injection: 20 μL. The counter-ion modifying agent triethylamine was added to obtain enhanced peak symmetry and minimize tailing. Furthermore, due to the large difference in the ratio of ATM to LUM (1:6), efforts were made to add a detectable amount of ATM without unnecessarily overloading the column with high concentrations of LUM. Sample solutions of the tablets were prepared by accurately weighing 4 mg of the powdered dosage form weighed into a clean dry beaker. 1 mL of acetic acid was added, allowed to react for a few minutes after which 5 mL of the mobile phase was added. The mixture was then shaken for 15 minutes on an ultrasonic sonicator, filtered into a 10 mL volumetric flask through a 0.45 μm filter, and made up to the mark through the filter with the mobile phase.

#### Assay for dihydroartemisinin in DHA/SP and DHA/Pp samples

A method outlined in the Ph. Int. [12] was modified as follows: column measurements: Kramasil C8, 25 cm x 4.6 mm, 5 μm; mobile phase: 50:50 v/v, water: acetonitrile; retention time (average): 5.2 minutes; detection wavelength: 210 nm*;* flow rate: 1.5 mL/min.; volume of injection: 10 μL. For each sample, a quantity of powdered tablets equivalent to 10 mg of DHA was accurately weighed into a clean dry beaker. This was extracted four times with diethyl ether as SP and Pp are practically insoluble in diethyl ether. This solution was evaporated to dryness. The residue was re-dissolved in 5 mL of the mobile phase, sonicated for 15 minutes, filtered through a 0.45 μm filter into a 10 mL volumetric flask and made up to the mark.

#### Assay for quinine in QN samples

QN was assayed according to a modified version in USP 24 [20]. Column measurements: Discovery C-18 bonded, 25 cm x 4.0 mm, 5 μm; mobile phase: 80:16:2:2 v/v, water: acetonitrile: methanesulfonic acid: TEA, pH 2.6; retention time (average): 5.7 minutes; detection wavelength: 235 nm*;* flow rate: 1.2 mL/min.; volume of injection: 20 μL. For the suspensions and mixtures, a certain amount was sonicated for about 15 minutes and a quantity equivalent to 5 mg was pipetted into a 10 ml volumetric flask. Methanol (8 ml) was added to the contents of the flask and made up to the mark with the mobile phase. Where necessary, the solution was also filtered.

For the injections, a 10 mL solution was prepared in a volumetric flask after 5 ampoules of quinine injections were mixed together and 20 μL aliquot was measured from the stock quinine solution using a microlitre syringe. The prepared solution was diluted with 8 mL of methanol and the mobile phase was added to the 10 mL mark.

#### Assay for sulphadoxine/sulphamethoxypyridazine and pyrimethamine

The experimental conditions employed in the analysis of S, Sm and P samples, were modified after a WHO-adopted monograph for inclusion into the Ph. Int. in 2010 [21]. Column measurements: Ascentis C-18, 15 cm x 4.60 mm, 5 μm; mobile phase: 65:10:25 v/v, 20 mM buffer (KH_2_PO_4_/Na_2_HPO_4_ of pH 5.6; methanol; acetonitrile; retention time (average): sulphadoxine/sulphamethoxypyridazine 3.9 minutes, pyrimethamine 8.7 minutes; detection wavelength: 240 nm; flow rate: 1 mL/min.; volume of injection: 10 μL. Solutions of sulphadoxine/sulphamethoxypyridazine and pyrimethamine containing tablets were prepared as follows: a quantity of the powdered dosage form equivalent to 100 mg of sulphadoxine/sulphamethoxypyridazine and 5 mg of pyrimethamine were weighed simultaneously into a clean dry beaker. The APIs were extracted three times for completeness using acetonitrile and finally made up to the mark with the mobile phase in a 50 mL volumetric flask.

#### Validation

Accuracy, precision, linearity and specificity parameters were evaluated for all the various determinations. Accuracy of results of an analytical method can also be established when validation parameters including precision (RSD values), linearity (R^2^ values), accuracy (% recovery) and specificity (retention times) were evaluated for all the various determinations (n = 6).

### Data interpretation

Poor quality medicines may be degraded, substandard or counterfeit. According to the WHO, Spurious/Falsely-Labelled/ Falsified/Counterfeit (SFFC) medicines (branded or generic) can be classified as “any medicines or pharmaceutical products that are deliberately and fraudulently mislabelled for identity and/or source” The definition includes products with correct or wrong ingredients, without active ingredients, with insufficient active ingredients, or with false packaging [22]*.* Substandard medicines, also known as Out of Specification (OOS) products are those that are genuine and legally produced but fall outside the specifications or acceptance criteria established in product dossiers, drug master files, pharmacopoeias or by the manufacturer. A degraded medicine can be classified along with substandard medicine. However, they differ in that they might be originally of specification, but in the course of time naturally or catalysed by external factors, fall out of specification within its shelf-life [5].

In this study, a sample was considered to have failed the quality evaluation if it did not meet any of the following criteria: 1) failure of visual inspection of dosage form and packaging, 2) failure to produce the expected colour reaction in the basic test and 3) failure to produce the expected spot colour or R_f_ on TLC compared to the reference standard. With respect to API content, a component API was classified as “compliant (C)” if its quantity fell within the acceptable International Pharmacopoeia limits of 90-110% of the amount of API stated on the label claim; “non-compliant (NC)” if the quantity was less than the lower (insufficient) or more than upper (excessive) acceptable limits [11,12]. Thus an ACT was considered compliant or to have passed the API content test only when both or all of its component APIs were compliant.

## Results

### Sample description

The anti-malarial samples analysed were 112, comprising 36 non-ACT, 4 ACT of single dose medicines co-packed on the same blister to be taken concomitantly and 72 ACT of fixed dose combinations. Artemether-lumefantrine (ATM/LUM) tablets, being the main ACT and serving as the first-line treatment for malaria in the country, formed the bulk of samples (36.6%) while SP tablets represented 20.5%. The formulations also included suspensions, injections and mixtures containing other APIs such as quinine (QN), piperaquine (Pp), sulphamethoxypyridazine (Sm), artesunate (ATS) and dihydroartemisinin (DHA). A total of 153 samples were collected, but the number analysed was limited by available assays and reference standards (RS). Table 1 shows a summary of the samples collected and analysed.

**Table 1:** **Categories of collected anti-malarial medicine samples**

**Non-ACT**		**ACT co-packed on one blister**		**Fixed dose ACT**	
**API**	**No.**	**API**	**No.**	**API**	**No.**
Quinine sulphate	6	ATS co-packed with SP	4	ATM/LUM	41
Quinine hydrochloride	3			DHA/Pp	14
Quinine bisulphate	4			DHA/SP	12
SP	23			ATS/SmP	5
**TOTAL**	**36**		**4**		**72**

**ATM**, artemether; **ATS** artesunate; **LUM**, lumefantrine; **DHA**, dihydroartemisinin; **S**, sulphadoxine; **P**, pyrimethamine; **Pp**, piperaquine phosphate; **Sm**, sulphamethoxypyridazine.

### Registration status of samples

The samples were subjected to registration verification with the Pharmacy, Medicine and Poisons Board of Malawi as soon as the collection exercise was completed. As of 31st December, 2011, 86.6% (97/112) of the collected samples as well as their formulation types were registered with the regulatory board, according to their annual registration publication. None of the samples was manufactured locally; all of them were imported samples with 60.7% manufactured in India and the rest originating from China, Kenya and Tanzania.

### Visual inspection of dosage form and packaging

The visual inspection of the dosage forms and packaging showed total (100%) compliance of the samples with the requirements. Labelling information regarding dosage form, brand name, active ingredient/strength, batch number, manufacture and expiry dates were provided (see Additional file 1). Tablets did not present with non-uniform colouration or signs of breakage.

### Basic tests

API content claims were confirmed by colourimetric tests, which demonstrated that all the samples contained the requisite APIs claimed by the manufacturers. However, it has been reported that with the advancement of counterfeiting, visual inspection and basic tests alone cannot qualify a medicine as genuine despite chemical and physical similarities. According to Bate *et al.*, the determination of a medicine as counterfeit or substandard requires a forensic examination of the trademarks; product designs and holograms [23], but the present study did not go as far as that.

### HPLC assay

**Figure 1:**
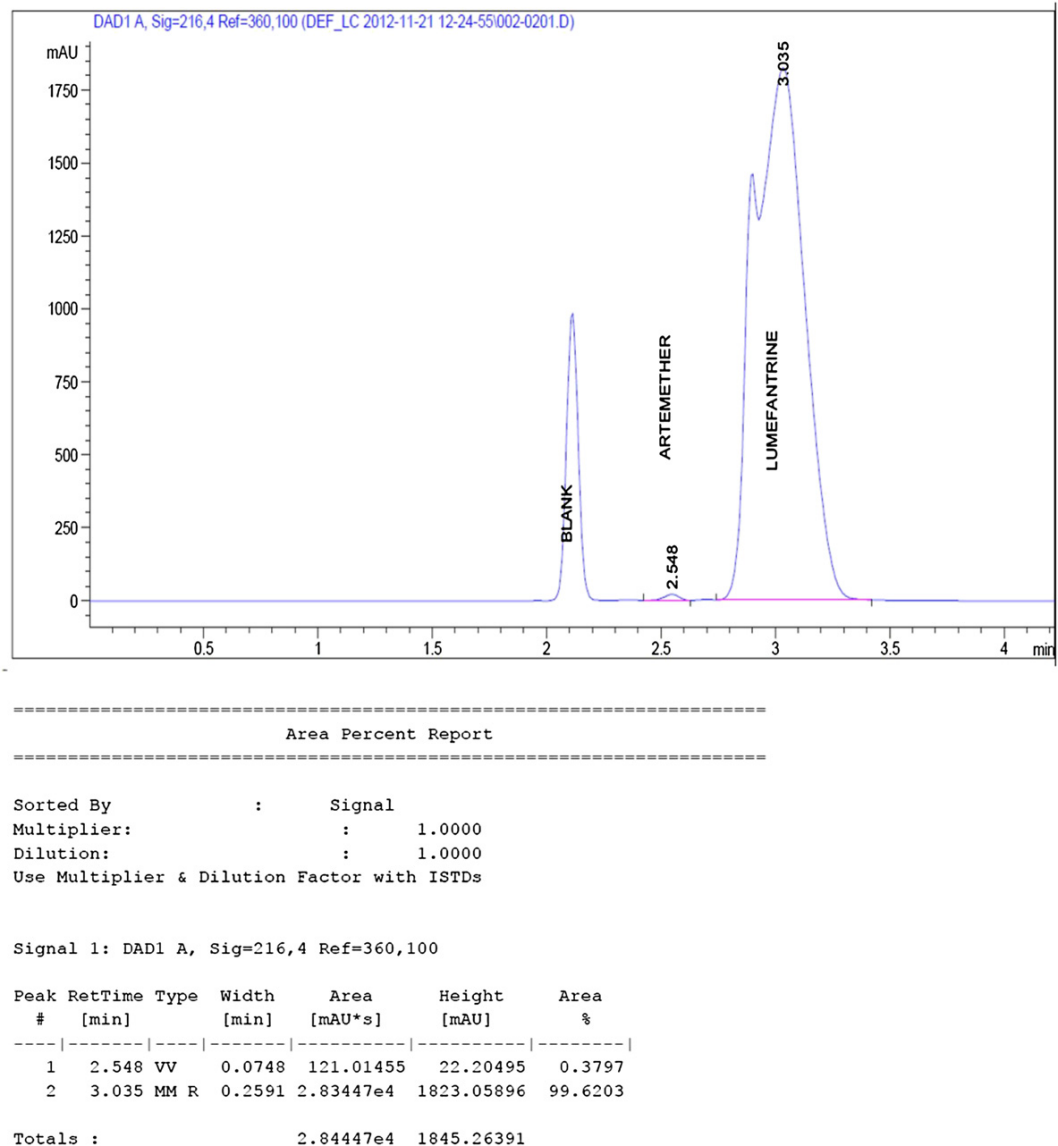
**Chromatogram of a 0.3 mg/mL and 1.7 mg/mL of ATM and LUM RS solutions.**

**Figure 2:**
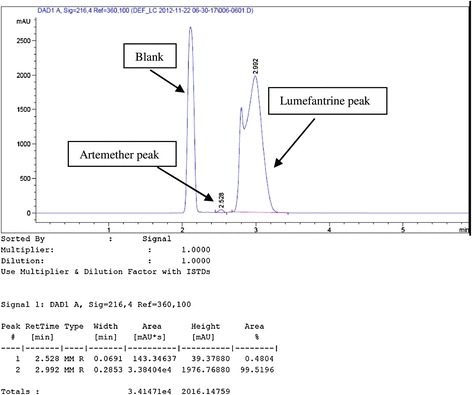
**Chromatogram for ATM/LUM tablet.**

**Figure 3:**
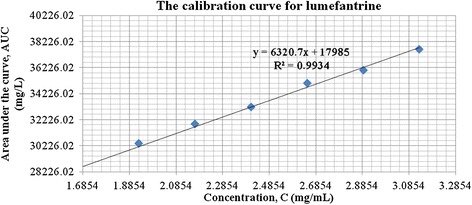
**Calibration curve for lumefantrine.**

**Figure 4:**
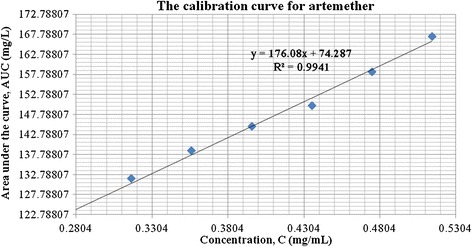
**Calibration curve for artemether.**

A major challenge in the simultaneous assay of ATM/LUM tablets was the choice of a solvent that would not interfere with the analyte peaks, dissolve both APIs long enough for the analysis to be carried out and also give well-resolved peaks. This was overcome by using acetic acid followed by acetonitrile to extract the active ingredients. From the chromatograms, acetic acid eluted first and did not interfere with the analyte peaks. Acetonitrile, with its low cut-off wavelength also did not interfere. The addition of the modifying agent triethylamine greatly enhanced peak symmetry and minimized tailing. A small shoulder appeared in the LUM peak and was observed in both the RS and the samples. Its presence did not hinder the computation of the AUC and was attributed to the column type, the mobile phase composition or both (Figures 1 and 2). Another challenge encountered was the difficulty in detecting artemether due to its low molar absorptivity and its low concentration (16.7%) in the fixed dose combination. Thus in the preparation of the calibration curves, different LUM concentrations were tried to obtain a range that would allow for the suitable detection of ATM and at the same time, not correspond to too high concentrations of LUM. The curves obtained were linear with R^2^ values of 0.994 for ATM and 0.993 for LUM (Figures 3 and 4).

The assay of SP samples was also problematic with respect to the high ratio of sulphadoxine to pyrimethamine, 20:1 respectively. Suitably high concentrations of sulfadoxine API were used to aid in the detection of pyrimethamine. The calibration curves obtained were linear with an R^2^ value of 0.999 and 0.998 for sulfadoxine and pyrimethamine respectively (Table 2). Details and sample chromatograms of all samples assayed together with their corresponding calibration curves are presented in Additional files 3 and 4.

**Table 2:** **Results of analytical method validation**

**Validation parameter**	**ATM**	**ATS**	**DHA**	**LUM**	**QN**	**S**	**P**
Precision (RSD)	0.836023149	0.088086277	1.165330278	0.896337033	2.00605798	1.77262931	0.0030604
Linearity(R^2^)	0.9940	0.9941	0.9950	0.9934	0.9979	0.9993	0.9975
Slope	176	502	410.1	6320	20180	16929	16600
Intercept	74.28	52.65	31.35	17985	162.9	4894	948.09
Specificity (Retention time)							
Medicine Sample API	2.528	5.081	5.225	2.992	6.113	3.928	8.689
Reference Standard	2.548	5.091	5.225	3.035	6.113	3.928	8.689

#### Validation

The accuracy of results of an analytical method can be established when validation parameters such as precision, linearity and specificity are clearly demonstrated. A summary of the method validation results is presented in Table 2, with details in Additional file 4. The average RSD values are ≤ 2%. The linearity values (R^2^) are also above 0.95 demonstrating a very good correlation between the peak area (AUC) and the concentration of the analyte APIs, linear across the 80-120% RS concentrations. The retention times of the analyte in the sample and the RS are also comparable, demonstrating high specificity.

#### Quality of anti-malarial medicines

Although all the samples passed the visual inspection and qualitative determination tests, the HPLC assay revealed that 88.4% (99/112) did not meet the requirements for API content (Table 3). The main cause of the failure was either the presence of insufficient API (i.e. < 90%) or excessive API (>110%). See Additional file 3 for detailed results.

**Table 3:** **Level of compliance of individual API content and failing rates of anti-malarial samples**

**Anti-malarial sample**	**Number of samples**	**Level of compliance of API content**			**Remarks**	**Number and rate of failing samples**
		**API**	**compliant**	**noncompliant**		
ATS/SP	4	ATS	4	0	All the 4 samples were noncompliant	4 (100%)
		S	0	4		
		P	2	2		
ATS/SmP	5	ATS	2	3	Compliant APIs did not occur in the same samples therefore all the samples were noncompliant	5 (100%)
		Sm	1	4		
		P	2	3		
ATM/LUM	41	ATM	14	27	Only 2 samples had both APIs being compliant in the same sample.	39 (95.1%)
		LUM	11	30		
DHA/Pp	14	DHA	4	10	Pp API could not be assayed. Based on DHA alone, 4 out of 14 samples were compliant	10 (71.4%)
		Pp	-	-		
DHA/SP	12	DHA	0	12	All the samples were noncompliant even though P was 100% compliant	12 (100%)
		S	1	11		
		P	12	0		
SP	23	S	3	20	Only 2 samples had both APIs being compliant in the same sample	21 (91.3%)
		P	12	11		
QN	13	QN	5	8	5 out 13 samples were compliant	8 (61.5%)
**Total**	**112**		**73**	**145**		**99 (88.4%)**

The 112 anti-malarial samples consisted of 9 APIs in various combinations with the exception of QN, which occurred as a monotherapy. Pp could not be assayed due to unavailability of a reference standard. Out of the 4 ATS/SP samples, ATS was compliant in all, P was compliant in 2 samples while S was compliant in none. The total noncompliance of the S component thus resulted in a 100% failure of all the ATS/SP samples.

ATS was compliant in only 2 out of the 5 ATS/SmP samples while Sm and P were compliant in 1 and 2 samples respectively. Regardless of having at least one case of compliance for each constituent API, this did not occur in the same sample. Hence, there was not a single sample in which all the individual APIs were compliant, resulting in 100% failure. In the 23 SP samples, compliance for S and P was in 3 and 12 samples respectively. Compliance for both constituents occurred together in only 2 samples resulting in 91.3% failure rate. There were 12 DHA/SP samples and although API content test for P was 100% compliant, 11 samples failed the S API content test while DHA was 100% noncompliant. Hence, no DHA/SP sample passed the quality test. Since Pp API in DHA/Pp could not be assayed, based on DHA alone, 4 out of the 14 samples were compliant (71.4% failure rate). In the case of the 41 ATM/LUM samples, only 2 samples had both APIs being compliant in the same sample. Overall, ATM was compliant in 14 samples while LUM was compliant in 11 samples. The failure rate of the samples was 91.5%. In the 13 QN monotherapy, 61.5% of the samples failed the quality test.

#### Failure rate of anti-malarial medicines versus registration status

**Figure 5:**
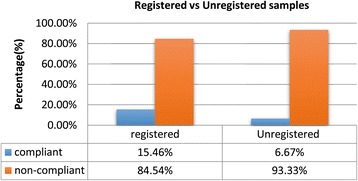
**A comparison of failure rates for registered and unregistered samples.**

Comparison of the failure rate of registered and unregistered samples suggested that registration status did not have significant influence on the quality of the anti-malarial medicines. Out of the 97 registered samples, 15.5% (15) of the samples were compliant, while 6.7% (1) of the 15 unregistered samples were compliant. Although the registered samples had a slight edge over the unregistered ones, the overall results (Figure 5) show that registration status does not necessarily always guarantee the quality a drug. These results are similar to the observations made in the 2008/2009 Ghana study [16].

#### Samples with the same batch number

API content of samples within same batches was compared to ascertain their uniformity. Some of the batch numbers were unregistered while others were duly registered. The results (Table 4) revealed wide variations in API content within batches, regardless of registration status.

**Table 4:** **Samples with the same batch number and their respective API content**

**Anti-malarial sample showing API and strength**	**Batch No.**	**Samples with the same batch number and their respective API content with HPLC assay**						
SDX/PYR	S-60	1_4_P_10_*	1_6_P_10_*	1_1_P_10_*	3_1_P_10_*	3_2_P_10_*	3_3_P_10_*	
500/25 mg		425/28 mg	415/23 mg	390/23 mg	415/21 mg	405/24 mg	410/22 mg	
SDX/PYR	1001	1_1_P_2_	1_4_P_2_	2_1_P_2_	3_5_P_2_			
500/25 mg	1	435/29 mg	410/23 mg	385/22 mg	265/12 mg			
SDX/PYR	1000	3_1_P_2_	4_4_P_2_	1_2_P_2_				
500/25 mg	8	485/24 mg	426/26 mg	335/19 mg				
QUN Bi-SO_4_	OK	1_2_ V_5_	4 V_5_					
50 mg/5 ml	159	60 mg	56 mg					
ATM/LUM	LD-	1_12_X_1_	1_5_X_1_	1_7_X_1_				
80/480 mg	266	81/492 mg	54/532 mg	65/283 mg				
ATM/LUM	LD-	1_9_X_1_	1_13_X_1_	1_10_X_1_	2_2_X_1_	4_4_X_1_		
80/480 mg	259	83/545 mg	112/528 mg	99/302 mg	105/302 mg	114/499 mg		
ATM/LUM	LF-	1_4_X_1_	1_6_X_1_	3_3_X_1_	4_3_X_1_			
40/240 mg	249	28/276 mg	65/305 mg	32/259 mg	70/196 mg			
ATM/LUM	LN-	1_8_X_1_	2_1_X_1_					
20/120 mg	450	33/95 mg	19/136 mg					
ATM/LUM	LS-	1_2_X_1_	3_1_X_1_	3_2_X_1_				
20/120 mg	39	24/136 mg	18/140 mg	19/140 mg				
ATM/LUM	SB01	1_2_X_11_	1_1_X_11_					
180/1080 mg	00I	186/1328 mg	167/1372 mg					
ATM/LUM	C052	3_1_X_11_	1_3_X_11_					
80/480 mg	0 J	31/569 mg	32/408 mg					
ATM/LUM	1105	1_2_X_14_	1_1_X_14_	2_4_X_14_				
20/120 mg	062	7/114 mg	15/83 mg	11/141 mg				
DHA/Pp	PX-	1_7_Z_1_	1_6_Z_1_	2_6_Z_1_				
40/320 mg	161	34/-	39/-	41/-				
DHA/SDX/PYR	AP-18	1_5_Z_1_	1_3_Z_1_	1_2_Z_1_	2_5_Z_1_	2_1_Z_1_	3_4_Z_1_	4_5_Z_1_
60/500/25 mg		51/428/24 mg	34/430/23 mg	32/414/25 mg	31/434/23 mg	34/385/26 mg	31/427/25 mg	31/438/26 mg
DHA/Pp	1101	1_4_Z_3_*	1_2_Z_3_*	1_1_Z_3_*	3_3_Z_3_*	4_4_Z_3_*	4_3_Z_3_*	4_1_Z_3_*
40/320 mg	23	28 mg/-	34 mg/-	39 mg/-	28 mg/-	35 mg/-	28 mg/-	30 mg/-
DHA/SDX/PYR	AP-	2_4_Z_1_	2_3_Z_1_	3_5_Z_1_				
	16	32/400/24 mg	33/450/25 mg	31/425/25 mg				
60/500/25 mg								
SDX/PYR	1001	2_2_P_2_	2_3_P_2_	3_2_P_2_	4_2_P_2_	4_5_P_2_		
500/25 mg	2	415/16 mg	480/31 mg	425/24 mg	235/13 mg	426/27 mg		
SDX/PYR	0213	2_1_P_15_	3_7_P_15_	3_8_P_15_				
500/25 mg	50	410/23 mg	355/20 mg	459/26 mg				
ATM/LUM	LD-	3_4_X_1_	3_6_X_1_					
80/480 mg	227	82/536 mg	84/496 mg					
ATS/SMP/PYR	081	3_1_Y_12_	4_1_Y_12_	4_2_Y_12_				
200/500/25 mg		178/217/14 mg	174/440/25 mg	186/443/29 mg				
ATS/SDX/PYR	TR0458	3_2_Y_13_*	3_4_Y_13_*					
100/500/25 mg		98/340/20 mg	98/370/18 mg					
QUN di-HCl	L-491	3_1_Q_6_	3_3_Q_6_	4_1_Q_6_	4_2_Q_6_			
150 mg/5 ml		166 mg	183 mg	153 mg	84 mg			
QUN HCl	110433	4_2_R_4_	4_3_R_4_					
100 mg/5 ml		151 mg	156 mg					
ATS/SMP/PYR	079	4_3_Y_12_	4_4_Y_12_					
100/250/12.5 mg		78/218/14 mg	90/225/15 mg					

* Unregistered samples.

#### Reporting of results

The findings of the study have not yet been made available to any of the regulatory bodies - the Pharmacy, Medicines and Poisons Board, the National Health Sciences Research Committee (NHSRC) of Malawi or the WHO Rapid Alert System.

## Discussion

The study showed a good correlation between both visual inspection of dosage form and packaging material on one hand and qualitative determination (basic tests) of API on the other because the labelled contents on the packaging materials were found to be correct. Thus, none of the samples can be considered as falsified with respect to visual inspection and qualitative determination. However, the quantitative assay revealed noncompliance of API content in a significant majority of the samples. There were 145 instances of the presence of a noncompliant API in the various categories of the anti-malarial medicines against 73 cases of compliance. In the combination formulations, more often than not, compliant APIs did not occur together in the same sample, resulting in the observed high failure rate in the quality evaluation (88.4%). Although the presence of insufficient API was the main cause of failing samples, there were cases of the presence of excessive API. In two previous studies on anti-malarial samples distributed in Ghana, where low quantities of API was also identified as a major contributory factor in failing rates, the artemisinin-based components of ACT were the insufficient APIs. Thus it was deduced that manufacturers could deliberately or otherwise be reducing quantities of the more expensive API as a means of cutting down on production cost. However, in the current quality evaluation, the artemisinin-based components have been detected in excessive quantities as well. This observation suggests poor adherence to SOPs, GMPs and proper registration procedures and is corroborated by the wide differences in API quantities of samples within batches (Table 4). In either case, there is the danger of sub-therapeutic doses of the noncompliant component promoting resistance or in the case where this component is present in excessive doses, posing a risk of toxicity to patients.

Although the registration status of anti-malarials used in Malawi was found to be quite satisfactory (13.4% unregistered), compared to countries such as Ghana and Togo where most recent studies indicated 55% and 78% unregistered anti-malarials in Ghana in 2008 and 2012 [16,17] respectively and 17% unregistered anti-malarials in Togo in 2012 [17], it has been established that registration of a medicine with the national regulatory authority does not necessarily guarantee its quality. ATM/LUM, SP and QN are the most commonly used medicines against malaria in Malawi, a country burdened with high transmission rate of malaria. Hence, the failure rate of these important medicines - ATM/LUM (95.1%), SP (91.3%) and QN (61.5%) - is alarming considering the fact most of the malaria cases in Malawi are diagnosed without microscopic determination. Most types of fever are presumed to be malaria first, and treated as such. If indeed, such ad hoc diagnoses are also treated with substandard anti-malarials, this could lead to treatment failure and/or fast development of resistance. This inference is based on the report by Bate *et al.* that resistance development of chloroquine and sulphadoxine in Africa in the 1990s and the devastating impact of malaria on the people were partly due to the use of substandard medicines [23].

### Comparison of current results with recent results from other African countries

Recent surveys on the quality of medicines circulating in many African countries have shown similar trends of poor quality anti-malarial medicines [7,16,17,24]*.* Most regulated manufacturers bypassed their GMP compliance and set the standards of their medicine products based on the recipient countries’ status with regard to the level of regulation capability and level of income as well as lack of prequalified standards by most developing countries to their suppliers [25]. Malawi, being a developing country and one of the poorest for that matter, is bound to suffer from poor regulatory capability and lack of expertise in routine rigorous medicine testing, considering the heavy reliance on imported anti-malarials. Most international surveys have rarely included samples from Malawi and efforts to locate any such internal activity at the required level have so far proved futile.

## Conclusions

The findings of the study suggest a widespread use of substandard anti-malarial medicines throughout the country with respect to API content. The detection of indiscriminate cases of excessive as well as insufficient API in both artemisinin-based and non-artemisinin-based components can be attributed to improper GMP and lack of quality control in the distribution chain. Therefore, there is an urgent need for regular rigorous testing by the National Medicines Regulatory Authority to deter importers from flooding the markets with poor quality medicines. Despite the effort put in place by the Government and its partners to minimize the impact of malaria over the years, the disease remains the country’s biggest health challenge. The post-marketing surveillance and pharmacovigilance system has been under development since 2009 and yet, it is still faced with severe limitations.

## Additional files

Additional file 1:
**List and details of anti-malarial medicines purchased.**
Additional file 2: Figure S1. Map of Malawi showing sampling sites. Additional file 3:
**Results of HPLC assay for API content in anti-malarial medicines.**
Additional file 4:
**Detailed analytical results.**

## Abbreviations

API: Active pharmaceutical ingredients
ACT: Artemisinin-based combination therapy
ATM: Artemether
ATS: Artesunate
C: Compliant
DHA: Dihydroartemisinin
EA: Enumeration area
EDQM: European Directorate for the Quality of Medicines and Healthcare
FDC: Fixed dose combination therapy
GMP: Good manufacturing practice
HPLC: High pressure liquid chromatography
LUM: Lumefantrine
NC: Non-compliant
NMCP: National Malaria Control Programme
P: Pyrimethamine
Ph. Int.: International pharmacopoeia
Pp: Piperaquine
PPS: Probability proportionality to size
QN: Quinine
RS: Reference standard
S: Sulphadoxine
SM: Sulphamethoxypyridazine
SOP: Standard operating procedure
SP: Sulphadoxine-pyrimethamine
SQ-TLC: Semi-quantitative thin layer chromatography
SRS: Simple random sampling
WHO: World Health Organization.

## References

[1] Butler AR, Khan S, Ferguson E (2010). A brief history of malaria chemotherapy. J R Coll Physicians Edinb.

[2] WHO (2014). World Malaria Report 2014.

[3] Programme NMC (2011). NMCP: Malaria strategic plan 2011–2015; towards universal access.

[4] Ettling M, McFarland DA, Schultz LJ, Chitsulo L (1994). Economic impact of malaria in Malawian households. Trop Med Parasitol.

[5] Dondorp AM, Yeung S, White L, Nguon C, Day NPJ, Socheat D (2010). Artemisinin resistance: current status and scenarios for containment. Nat Rev Microbiol.

[6] Newton PN, White NJ, Rozendaal JA, Green MD (2002). Murder by fake medicines. BMJ.

[7] Nayyar GML, Breman JG, Newton PN, Herrington J (2012). Poor-quality antimalarial, medicines in southeast Asia and sub-Saharan Africa. Lancet Infect Dis.

[8] Maponga C, Ondari C. The quality of antimalarials; a study in selected African countries. World Health Organization (WHO) Department of Essential Medicines and Medicines. WHO/EDM/PAR/2003.4; 2003 [http://apps.who.int/medicinedocs/pdf/s4901e/s4901e.pdf]

[9] Staedke S: Medicine safety and quality. London School of Hygiene and Tropical Medicine; Uganda Malaria Surveillance Project. ACT Consortium; 2009. [http://www.actconsortium.org/data/files/actc_safety_and_quality__overview.pdf]

[10] Strengthening Pharmaceutical Systems (SPS) Program: Safety of medicines in sub-Saharan Africa: assessment of pharmacovigilance systems and their performance. US Agency for International Development by the Strengthening Pharmaceutical Systems (SPS) Program. Arlington, VA: Management Sciences for Health; 2011. [http://apps.who.int/medicinedocs/en/d/Js19152en/]

[11] World Health Organization: The International pharmacopoeia (including first, second and third supplements). 4th Edition, Version 2; Online, Geneva; 2013. [http://apps.who.int/phint/en/p/about/]

[12] World Health Organization (2006). The International pharmacopoeia 4th Edition, Version 2; CD-ROM, Geneva.

[13] World Health Organization, BTD. Basic tests for medicines: pharmaceutical substances, medicinal plant materials and dosage form; 1998. [http://whqlibdoc.who.int/publications/1998/9241545135.pdf]

[14] World Health Organization (2006). New basic tests for antimalarials.

[15] WHO: Basic tests for pharmaceutical dosage forms. Geneva; World Health Organization, 1991. [http://apps.who.int/medicinedocs/pdf/h1794e/h1794e.pdf]

[16] Osei-Safo D, Harrison JJEK, Addae-Mensah I: Validation and application of quality assurance methods developed for artemisinin-based antimalarial medicines to assess the quality of a selection of such medicines distributed in Accra, Ghana. African Journal of Pharmaceutical Sciences and Pharmacy 2010, 1: 1–25. Retrieved from [http://www.ajpspjournal.com/article/view/6206]

[17] Osei-Safo D, Agbonon A, Konadu DY, Harrison JJEK, Edoh M, Gordon A, Gbeassor M, Addae-Mensah I: Evaluation of the quality of artemisinin-based antimalarial medicines distributed in Ghana and Togo. Malar Res Treat 2014, Article ID 806416, 12 pages. [http://www.hindawi.com/journals/mrt/2014/806416/] 10.1155/2014/806416 PMC4225840 25400975

[18] Ranher SS, Gandhi SV, Kadukar SS, Ranjane PN (2010). A validated HPLC method for determination of artesunate in bulk and tablet formulation. J Anal Chem.

[19] Arun A, Smith AA (2011). Simultaneous HPLC-UV method for the estimation of artemether and lumefantrine in tablet dosage form. Int J Pharma Biomed Res.

[20] The United States Pharmacopoeia USP NF 24 (2006). Monographs of quinine sulphate and quinine sulphate tablets.

[21] WHO expert committee. Sulphadoxine and pyrimethamine tablets. Adopted text for addition to The International Pharmacopoeia. World Health Organization Working document QAS/07.218/FINAL; 2011. [http://www.who.int/medicines/publications/pharmacopoeia/Sulfadox-Pyrimeth-tab-QAS07-218FINAL.pdf]

[22] World Health Organization (1999). Counterfeit drugs. Guidelines for the development of measures to combat counterfeit drugs.

[23] Bate R, Coticelli P, Tren R, Attaran A (2008). Antimalarial medicine quality in the most severely malarious parts of Africa – A six country study. PLoS One.

[24] WHO QAMSA. Survey of the quality of selected antimalarial medicines circulating in six countries of sub-Saharan Africa. Geneva: World Health Organization Quality Assurance and Safety; Essential Medicines and pharmaceutical Policies, WHO/EMP/QSM/2011.1; 2011. [http://www.who.int/medicines/publications/WHO_QAMSA_report.pdf]

[25] Caudron JM, Ford N, Henkens M, Macé C, Kiddle-Monroe R, Pinel J (2008). Substandard medicines in resource-poor settings: a problem that can no longer be ignored. Trop Med Int Health.

