# Supplementary material for: Post-marketing surveillance of anti-malarial medicines used in Malawi
Source: Malar J. 2015 Mar 25;14:127. doi: 10.1186/s12936-015-0637-z (PMC4377194; doi:10.1186/s12936-015-0637-z)
Supplement: Additional file 1: — List and details of anti-malarial medicines purchased. [file 12936_2015_637_MOESM1_ESM.docx]

**LIST AND DETAILS OF ANTIMALARIAL DRUGS PURCHASED**

List of artemisinin-based and non-artemisinin based antimalarial drugs purchased from various zones in Malawi

| **No.** | **Code** | **Name of drug** | **Dosage form** | **Active ingredient** | **Batch no.** | **Manufacturer** | **Man–Exp**  **Date** |
| --- | --- | --- | --- | --- | --- | --- | --- |
| **ZONE 1: SOUTH WEST** | | | | | | | |
| **NON-ARTEMISININ-BASED DRUGS** | | | | | | | |
| 1 | *1_4_P_10_ | Malacure | Tablet | Sulphadoxine USP/ Pyrimethamine USP  500mg/ 25mg  fixed dose | S-60 | S Kant  India | 07/2010-  06/2013 |
| 2 | *1_6_P_10_ | Malacure | Tablet | Sulphadoxine USP/ Pyrimethamine USP  500mg/ 25mg  fixed dose | S-60 | S Kant  India | 07/2010-  06/2013 |
| 3 | *1_1_P_10_ | Malacure | Tablet | Sulphadoxine USP/ Pyrimethamine USP  500mg/ 25mg  fixed dose | S-60 | S Kant  India | 07/2010-  06/2013 |
| 4 | 1_1_P_2_ | Sulphadar | Tablet | Sulphadoxine USP/ Pyrimethamine USP  500mg/ 25mg  fixed dose | 10011 | Shellys  Tanzania | 07/2010-  06/2014 |
| * Unregistered samples | | | | | | | |
| 5 | 1_2_P_2_ | Sulphadar | Tablet | Sulphadoxine USP/ Pyrimethamine USP  500mg/ 25mg  fixed dose | 10008 | Shellys  Tanzania | 05/2010-  04/2014 |
| 6 | 1_4_P_2_ | Sulphadar | Tablet | Sulphadoxine USP/ Pyrimethamine USP  500mg/ 25mg  fixed dose | 10011 | Shellys  Tanzania | 07/2010-  06/2014 |
| 7 | 1_3_V_5_ | Quinaquin  100ml | Mixture | Quinine Bisulphate BP  50mg/5ml | OK 157 | Elys Chemical  Kenya | 11/2010-  10/2012 |
| 8 | 1_2_V_5_ | Quinaquin  100ml | Mixture | Quinine Bisulphate BP  50mg/5ml | OK 159 | Elys Chemical  Kenya | 11/2010-  10/2010 |
| 9 | 1_1_V_5_ | Quinaquin  100ml | Mixture | Quinine Bisulphate BP  50mg5/ml | OL 119 | Elys Chemical | 12/2010-  11/2012 |
| **ARTEMISININ-BASED DRUGS** | | | | | | | |
| 10 | 1_7_X_1_ | Lonart-DS | Tablet | Artemether/ Lumefantrine  80mg/480mg  fixed dose | LD-266 | Bliss GVS  India | 09/2011-  08/2013 |
| 11 | 1_9_X_1_ | Lonart-DS | Tablet | Artemether/ Lumefantrine  80mg/480mg  fixed dose | LD-259 | Bliss GVS  India | 08/2011-07/2013 |
| * Unregistered samples | | | | | | | |
| 12 | 1_13_X_1_ | Lonart-DS | Tablet | Artemether/ Lumefantrine  80mg/480mg  fixed dose | LD-259 | Bliss GVS  India | 08/2011-  07/2013 |
| 13 | 1_10_X_1_ | Lonart-DS | Tablet | Artemether/ Lumefantrine  80mg/480mg  fixed dose | LD-259 | Bliss GVS  India | 08/2011-07/2013 |
| 14 | 1_12_X_1_ | Lonart-DS | Tablet | Artemether/ Lumefantrine  80mg/480mg  fixed dose | LD-266 | Bliss GVS  India | 09/2011-  08/2013 |
| 15 | 1_5_X_1_ | Lonart-DS | Tablet | Artemether/ Lumefantrine  80mg/480mg  fixed dose | LD-266 | Bliss GVS  India | 09/2011-  08/2013 |
| 16 | 1_6_X_11_ | Lonart | Suspension | Artemether/ Lumefantrine  180mg/1080mg  fixed dose | LO-210 | Bliss GVS  India | 08/2011-  07/2013 |
| 17 | 1_4_X_1_ | Lonart Forte | Tablet | Artemether/ Lumefantrine  40mg/240mg  fixed dose | LF-249 | Bliss GVS  India | 08/2011-  07/2013 |
| 18 | 1_6_X_1_ | Lonart Forte | Tablet | Artemether/ Lumefantrine  40mg/240mg  fixed dose | LF-249 | Bliss GVS  India | 08/2011-  07/2013 |
| 19 | 1_3_X_1_ | Lonart Forte | Tablet | Artemether/ Lumefantrine  40mg/240mg  fixed dose | LF-239 | Bliss GVS  India | 05/2011-  04/2013 |
| 20 | 1_8_X_1_ | Lonart | Tablet | Artemether/ Lumefantrine  20mg/120mg  fixed dose | LN-450 | Bliss GVS  India | 09/2011-  08/2013 |
| 21 | 1_2_X_1_ | Lonart-Dispersible | Tablet | Artemether/ Lumefantrine  20mg/120mg  fixed dose | LS-39 | Bliss GVS  India | 09/2011-  08/2013 |
| 22 | 1_1_X_1_ | Lonart | Tablet | Artemether/ Lumefantrine  20mg/120mg  fixed dose | LN-449 | Bliss GVS  India | 09/2011-  08/2013 |
| * Unregistered samples | | | | | | | |
| 23 | 1_2_X_11_ | Artefan | Suspension | Artemether/ Lumefantrine  180mg/1080mg  fixed dose | SB0100I | Ajanta  India | 09/2010-  08/2012 |
| 24 | 1_1_X_11_ | Artefan | Suspension | Artemether/ Lumefantrine  180mg/1080mg  fixed dose | SB0100I | Ajanta  India | 09/2010-  08/2012 |
| 25 | 1_4_X_11_ | Artefan | Suspension | Artemether/ Lumefantrine  40mg/240mg  fixed dose | C0490J | Ajanta  India | 10/2010-09/2012 |
| 26 | 1_3_X_11_ | Artefan | Tablet | Artemether/ Lumefantrine  80mg/480mg  fixed dose | C0520J | Ajanta  India | 10/2010-  09/2012 |
| 27 | 1_5_X_11_ | Artefan | Tablet | Artemether/ Lumefantrine  20mg/120mg  fixed dose | C0480J | Ajanta  India | 10/2010-  09/2012 |
| 28 | 1_1_X_20_ | Coartem-Dispersible | Tablet | Artemether/ Lumefantrine  20mg/120mg  fixed dose | F0493 | Norvatis  USA | 08/2011-  07/2013 |
| 29 | 1_1_X_17_ | Fantem-Forte | Tablet | Artemether/ Lumefantrine  80mg/480mg  fixed dose | M110371 | Medinomics  India | 05/2011-  04/2013 |
| 30 | 1_2_X_14_ | Lum-Artem | Tablet | β-Artemether/ Lumefantrine  20mg/120mg  fixed dose | 1105062 | Dawa Ltd  Kenya | 05/2011-  04/2014 |
| 31 | 1_1_X_14_ | Lum-Artem | Tablet | β-Artemether/ Lumefantrine  20mg/120mg  fixed dose | 1105062 | Dawa Ltd  Kenya | 05/2011-  04/2014 |
| 32 | 1_1_X_18_ | LA-DS | Tablet | Artemether/ Lumefantrine  40mg/240mg  fixed dose | 1117 | Global Pharma  India | 04/2010-  03/2013 |
| 33 | 1_7_Z_1_ | P-Alaxin | Tablet | Dihydroartemisinin/Piperaquine Phosphate  40mg/320mg  fixed dose | PX-161 | Bliss GVS  India | 08/2011-  07/2014 |
| * Unregistered samples | | | | | | | |
| 34 | 1_6_Z_1_ | P-Alaxin | Tablet | Dihydroartemisinin/Piperaquine Phosphate  40mg/320mg  fixed dose | PX-161 | Bliss GVS  India | 08/2011-  07/2014 |
| 35 | 1_5_Z_1_ | Alaxin | Tablet | Dihydroartemisinin/Sulphadoxine BP/Pyrimethamine BP  60mg/500mg/25mg  fixed dose | AP-18 | Bliss GVS  India | 08/2011-  07/2014 |
| 36 | 1_4_Z_1_ | Alaxin | Tablet | Dihydroartemisinin/Sulphadoxine BP/Pyrimethamine BP  60mg/500mg/25mg  fixed dose | AP-17 | Bliss GVS  India | 03/2011-  02/2014 |
| 37 | 1_3_Z_1_ | Alaxin | Tablet | Dihydroartemisinin/Sulphadoxine BP/Pyrimethamine BP  60mg/500mg/25mg  fixed dose | AP-18 | Bliss GVS  India | 08/2011-  02/2014 |
| 38 | 1_2_Z_1_ | Alaxin | Tablet | Dihydroartemisinin/Sulphadoxine BP/Pyrimethamine BP  60mg/500mg/25mg  fixed dose | AP-18 | Bliss GVS  India | 08/2011-  02/2014 |
| 39 | *1_4_Z_3_ | Duo-Cotecxin | Tablet | Dihydroartemisinin/Piperaquine Phosphate  40mg/320mg  fixed dose | 110123 | Zhejiang Holley Nanhu  China | 01/2011-01/2013 |
| 40 | *1_2_Z_3_ | Duo-Cotecxin | Tablet | Dihydroartemisinin/Piperaquine Phosphate  40mg/320mg  fixed dose | 110123 | Zhejiang Holley Nanhu  China | 01/2011-01/2013 |
| 41 | *1_1_Z_3_ | Duo-Cotecxin | Tablet | Dihydroartemisinin/Piperaquine Phosphate  40mg/320mg  fixed dose | 110123 | Zhejiang Holley Nanhu  China | 01/2011-01/2013 |
| * Unregistered samples | | | | | | | |
| **ZONE 2: SOUTH EAST** | | | | | | | |
| **ARTEMISININ-BASED DRUGS** | | | | | | | |
| 42 | 2_3_X_1_ | Lonart | Tablet | Artemether/ Lumefantrine  20mg/120mg  fixed dose | LN-262 | Bliss GVS  India | 02/2010-  01/2012 |
| 43 | 2_1_X_1_ | Lonart | Tablet | Artemether/ Lumefantrine  20mg/120mg  fixed dose | LN-450 | Bliss GVS  India | 09/2011-  08/2013 |
| 44 | 2_2_X_1_ | Lonart | Tablet | Artemether/ Lumefantrine  80mg/480mg  fixed dose | LD-259 | Bliss GVS  India | 08/2011-  07/2013 |
| 45 | 2_4_X_14_ | Lum-Artem | Tablet | β-Artemether/ Lumefantrine  20mg/120mg  fixed dose | 1105062 | Dawa Ltd  Kenya | 05/2011-  04/2014 |
| 46 | 2X_15_ | Co-Max | Tablet | Artemether/ Lumefantrine  20mg/120mg  fixed dose | 021367 | Universal  Kenya | 08/2010-  07/2012 |
| 47 | 2_5_X_11_ | Artefan | Tablet | Artemether/ Lumefantrine  80mg/480mg  fixed dose | C0601A | Ajanta  India | 01/2011-  12/2012 |
| 48 | 2_4_Y_13_ | Spafil | Tablet | Artesunate/Sulphadoxin/Pyrimethamine 100/500/25mg  co-packed on the same blister | T0458 | Fourrts | 02/2011-  01/2014 |
| 49 | 2_5_Z_1_ | Alaxin | Tablet | Dihydroartemisinin/Sulphadoxine BP/Pyrimethamine BP  60mg/500mg/25mg  fixed dose | AP-18 | Bliss GVS  India | 08/2011-  07/2014 |
| 50 | 2_4_Z_1_ | Alaxin | Tablet | Dihydroartemisinin/Sulphadoxine BP/Pyrimethamine BP  60mg/500mg/25mg  fixed dose | AP-16 | Bliss GVS  India | 02/2011-  01/2014 |
| * Unregistered samples | | | | | | | |
| 51 | 2_1_Z_1_ | Alaxin | Tablet | Dihydroartemisinin/Sulphadoxine BP /Pyrimethamine BP  60mg/500mg/25mg  fixed dose | AP-18 | Bliss GVS  India | 08/2011-  07/2014 |
| 52 | 2_3_Z_1_ | Alaxin | Tablet | Dihydroartemisinin/Suphfadoxine BP /Pyrimethamine BP  60mg/500mg/25mg  fixed dose | AP-16 | Bliss GVS  India | 02/2011-  01/2014 |
| 53 | 2_6_Z_1_ | P-Alaxin | Tablet | Dihydroartemisinin/Piperaquine Phosphate 40/320mg | PX-161 | Bliss GVS  India | 08/2011-  07/2014 |
| 54 | 2_7_Z_1_ | P-Alaxin | Tablet | Dihydroartemisinin/Piperaquine Phosphate 40/320mg | PX-116 | Bliss GVS  India | 02/2011-01/2014 |
| **NON-ARTEMISININ BASED DRUGS** | | | | | | | |
| 55 | 2_1_P_2_ | Sulphadar | Tablet | Sulphadoxine USP /Pyrimethamine USP  500mg/ 25mg  fixed dose | 10011 | Shellys  Tanzania | 07/2010-  06/2014 |
| 56 | 2_2_P_2_ | Sulphadar | Tablet | Sulphadoxine USP /Pyrimethamine USP  500mg/ 25mg  fixed dose | 10012 | Shellys  Tanzania | 07/2010-  06/2014 |
| 57 | 2_3_P_2_ | Sulphadar | Tablet | Sulphadoxine USP /Pyrimethamine USP  500mg/ 25mg  fixed dose | 10012 | Shellys  Tanzania | 07/2010-  06/2014 |
| 58 | 2_1_P_15_ | Methomine S | Tablet | Sulphadoxine BP /Pyrimethamine BP  500mg/25mg  fixed dose | 021350 | Universal  Kenya | 08/2010-  07/2013 |
| * Unregistered samples | | | | | | | |
| **ZONE 3 : CENTRAL** | | | | | | | |
| **ARTEMISININ-BASED DRUGS** | | | | | | | |
| 59 | 3_1_X_11_ | Artefan | Tablet | Artemether/Lumefantrine  80mg/480mg  fixed dose | C0520J | Ajanta  India | 10/2010-  09/2012 |
| 60 | 3_1_X_1_ | Lonart-Dispersible | Tablet | Artemether/ Lumefantrine  20mg/120mg  fixed dose | LS-39 | Bliss GVS  India. | 09/2011-  08/2013 |
| 61 | 3_2_X_1_ | Lonart-Dispersible | Tablet | Artemether/ Lumefantrine  20mg/120mg  fixed dose | LS-39 | Bliss GVS  India. | 09/2011-  08/2013 |
| 62 | 3_3_X_1_ | Lonart Forte | Tablet | Artemether/Lumefantrine  40mg/240mg  fixed dose | LF-249 | Bliss GVS  India | 08/2011-  07/2013 |
| 63 | 3_4_X_1_ | Lonart-DS | Tablet | Artemether/Lumefantrine  800mg/480mg  fixed dose | LD-227 | Bliss GVS  India | 05/2011-  04/2013 |
| 64 | 3_6_X_1_ | Lonart-DS | Tablet | Artemether/Lumefantrine  80mg/480mg  fixed dose | LD-227 | Bliss GVS  India | 05/2011-  04/2013 |
| 65 | 3_1_Y_12_ | Co-Arinate FDC(Adult) | Tablet | Artesunate/Sulphamethoxypyridazine/Pyrimethamine 200/500/25mg  fixed dose | 081 | Dafra  Kenya | 02/2011-  02/2013 |
| 66 | 3_2_Y_13_ | Spafil(Adults) | Tablet | Artesunate/Sulphadoxine/Pyrimethamine 100/500/25mg  co-packed on the same blister | TR0458 | Fourrts  India | 02/2011-  01/2014 |
| 67 | 3_4_Y_13_ | Spafil(Adults) | Tablet | Artesunate/Sulphadoxine/Pyrimethamine 100/500/25mg  co-packed on the same blister | TR0458 | Fourrts  India | 02/2011-  01/2014 |
| * Unregistered samples | | | | | | | |
| 68 | *3_2_Z_3_ | Duo-Cotecxin | Tablet | Dihydroartemisinin/Piperaquine Phosphate 40/320mg  fixed dose | 210610 | Zhejiang Holley Nanhu  China | 06/2010-  06/2012 |
| 69 | *3_3_Z_3_ | Duo-Cotecxin | Tablet | Dihydroartemisinin/Piperaquine Phosphate 40/320mg  fixed dose | 110123 | Zhejiang Holley Nanhu | 01/2011-  01/2013 |
| 70 | 3_4_Z_1_ | Alaxin +(plus) | Tablet | Dihydroartemisinin/Sulphadoxine BP/Pyrimethamine BP 60/500/25mg  fixed dose | AP-18 | Bliss GVS  India | 08/2011-  07/2014 |
| 71 | 3_5_Z_1_ | Alaxin +(plus) | Tablet | Dihydroartemisinin/Sulphadoxine BP/Pyrimethamine BP 60/500/25mg  fixed dose | AP-16 | Bliss GVS  India | 02/2011-  01/2014 |
| 72 | 3_7_Z_1_ | P-Alaxin | Tablet | Dihydroartemisinin/Piperaquine Phosphate 40/320mg  fixed dose | PX-146 | Bliss GVS  India | 06/2011-  05/2014 |
| **NON-ARTEMISININ BASED DRUGS** | | | | | | | |
| 73 | *3_1_P_10_ | Malacure | Tablet | Sulphadoxine/Pyrimethamine  500mg/25mg  fixed dose | S-60 | S Kant  Mumbai | 07/2010-  06/2013 |
| 74 | *3_2_P_10_ | Malacure | Tablet | Sulphadoxine/Pyrimethamine  500mg/25mg  fixed dose | S-60 | S Kant  Mumbai | 07/2010-  06/2013 |
| 75 | *3_3_P_10_ | Malacure | Tablet | Sulphadoxine/Pyrimethamine  500mg/25mg  fixed dose | S-60 | S Kant  Mumbai | 07/2010-  06/2013 |
| 76 | 3_1_P_2_ | Sulphadar | Tablet | Sulphadoxine USP/Pyrimethamine USP  500mg/25mg  fixed dose | 10008 | Shellys  Tanzania | 05/2010-  04/2014 |
| * Unregistered samples | | | | | | | |
| 77 | 3_2_P_2_ | Sulphadar | Tablet | Sulphadoxine USP/Pyrimethamine USP  500mg/25mg  fixed dose | 10012 | Shellys  Tanzania | 07/2010-  06/2014 |
| 78 | 3_5_P_2_ | Sulphadar | Tablet | Sulphadoxine USP/Pyrimethamine USP  500mg/25mg  fixed dose | 10011 | Shellys  Tanzania | 07/2010-  06/2014 |
| 79 | 3_7_P_15_ | Methomine S | Tablet | Sulphadoxine BP/Pyrimethamine BP  500mg/25mg  fixed dose | 021350 | Universal  Kenya | 08/2010-  07/2013 |
| 80 | 3_8_P_15_ | Methomine S | Tablet | Sulphadoxine BP/Pyrimethamine BP  500mg/25mg  fixed dose | 021350 | Universal  Kenya | 08/2010-  07/2013 |
| 81 | 3_1_Q_6_ | Quinine Sulphate Suspension  (QSM) | Suspension | Quinine Sulphate 150mg | L-491 | Lebene Laboratories  India | 06/2011-  05/2013 |
| 82 | 3_2_Q_6_ | Quinine Sulphate Suspension  (QSM) | Suspension | Quinine Sulphate 150mg | L-1180 | Lebene Laboratories  India | 08/2010-  07/2012 |
| 83 | 3_3_Q_6_ | Quinine Sulphate Suspension  (QSM) | Suspension | Quinine Sulphate 150mg | L-491 | Lebene Laboratories  India | 06/2011-  05/2013 |
| **ZONE 4 : NORTH** | | | | | | | |
| **NON-ARTEMISININ-BASED DRUGS** | | | | | | | |
| 84 | 4V_5_ | Quinaquin | Mixture | Quinine bisulphate BP 50mg/5ml | OK 159 | Elys Chemical  Kenya | 11/2010-  10/2012 |
| 85 | 4_1_R_8_ | Kwinil | Injection | Quinine di-HCl | L01224 | Intas Pharmaceuticals  India | 02/2010-  01/2013 |
| * Unregistered samples | | | | | | | |
| 86 | 4_1_Q_6_ | Quinine Sulphate Suspension  (QSM) | Suspension | Quinine sulphate 150mg/5ml | L-491 | Lebene  Laboratories  India | 06/2011-  05/2013 |
| 87 | 4_2_Q_6_ | Quinine Sulphate Suspension  (QSM) | Suspension | Quinine sulphate 150mg/5ml | L-491 | Lebene  Laboratories  India | 06/2011-  05/2013 |
| 88 | 4_3_Q_6_ | Quinine Sulphate Suspension  (QSM) | Suspension | Quinine sulphate 150mg/5ml | L-2100 | Lebene  Laboratories  India | 12/2010-  11/2012 |
| 89 | 4_2_R_4_ | Curaquin | Quinine syrup | Quinine HCl BP 100mg/5ml | 110433 | Regal Pharmaceuticals  Kenya | 04/2011-  03/2014 |
| 90 | 4_3_R_4_ | Curaquin | Quinine syrup | Quinine HCl BP 100mg/5ml | 110433 | Regal Pharmaceuticals  Kenya | 04/2011-  03/2014 |
| 91 | 4_1_P_2_ | Sulphadar | Tablet | Sulphadoxine USP/Pyrimethamine USP  500mg/25mg  fixed dose | 10013 | Shellys  Tanzania | 07/2010-  06/2014 |
| 92 | 4_4_P_2_ | Sulphadar | Tablet | Sulphadoxine USP/Pyrimethamine USP  500mg/25mg  fixed dose | 10008 | Shellys  Tanzania | 05/2010-  04/2014 |
| 93 | 4_2_P_2_ | Sulphadar | Tablet | Sulphadoxine USP/Pyrimethamine USP  500mg/25mg  fixed dose | 10012 | Shellys  Tanzania | 07/2010-  06/2014 |
| 94 | 4_5_P_2_ | Sulphadar | Tablet | Sulphadoxine USP/Pyrimethamine USP  500mg/25mg  fixed dose | 10012 | Shellys  Tanzania | 07/2010-  06/2014 |
| 95 | 4_8_P_5_ | Ekelfin | Tablet | Sulphadoxine USP/Pyrimethamine USP  500mg/25mg  fixed dose | OA 92 | Elys Chemical  Kenya | 01/2010-  12/2013 |
| * Unregistered samples | | | | | | | |
| **ARTEMISININ-BASED DRUGS** | | | | | | | |
| 96 | 4_5_X_12_ | Co-Artesiane | Suspension | Artemether/Lumefantrine 180mg:60ml/1080mg:60ml | 24243 | Dafra Pharma  Kenya | 06/2010-  06/2012 |
| 97 | 4X_20_ | Coartem-Dispersible  (Children) | Tablet | Artemether/Lumefantrine 20/120mg  fixed dose | F0442 | Novartis  USA | 05/2011-  04/2013 |
| 98 | 4_4_X_1_ | Lonart-DS | Tablet | Artemether/Lumefantrine 80/480mg  fixed dose | LD- 259 | Bliss Gvs  India | 08/2011-  07/2013 |
| 99 | 4_3_X_1_ | Lonart Forte | Tablet | Artemether + lumefantrine 80/480mg  fixed dose | LF-249 | Bliss Gvs  India | 08/2011-  07/2013 |
| 100 | 4_2_X_11_ | Artefan | Tablet | Artemether/Lumefantrine 20/120mg  fixed dose | C0411G | Ajanta  India | 07/2011-  06/2013 |
| 101 | 4_1_X_11_ | Artefan | Tablet | Artemether/Lumefantrine 20/120mg  fixed dose | P0761G | Ajanta  India | 07/2011-  06/2013 |
| 102 | 4Y_13_ | Spafil | Tablet | Artesunate/Sulphadoxine/Pyrimethamine 100/500/25mg  co-packed on the same blister | TR0324 | Fourrts  India | 05/2010-  04/2013 |
| 103 | 4_3_Y_12_ | Co-Arinate PDC | Tablet | Artesunate/Sulphamethoxypyridazine/Pyrimethamine 100/250/12.5mg  fixed dose | 079 | Dafra Pharma  Kenya | 03/2011-  03/2013 |
| 104 | 4_1_Y_12_ | Co-Arinate PDC | Tablet | Artesunate/Sulphamethoxypyridazine/Pyrimethamine 200/500/25mg  fixed dose | 081 | Dafra Pharma  Kenya | 02/2011-  02/2013 |
| 105 | 4_2_Y_12_ | Co-Arinate PDC | Tablet | Artesunate/Sulphamethoxypyridazine/Pyrimethamine 200/500/25mg | 081 | Dafra Pharma  Kenya | 02/2011-  02/2013 |
| 106 | 4_4_Y_12_ | C0-Arinate PDC | Tablet | Artesunate/Sulphamethoxypyridazine/Pyrimethamine 100/250/12.5mg | 079 | Dafra Pharma  Kenya | 03/2011-  03/2013 |
| 107 | *4_4_Z_3_ | Duo-Cotecxin | Tablet | Dihydroartemisinin/Piperaquine Phosphate 40/320mg  fixed dose | 110123 | Zhejiang Holley Nanhu  China | 01/2011-  01/2013 |
| * Unregistered samples | | | | | | | |
| 108 | *4_3_Z_3_ | Duo-Cotecxin | Tablet | Dihydroartemisinin/Piperaquine Phosphate 40/320mg  fixed dose | 110123 | Zhejiang Holley Nanhu  China | 01/2011-  01/2013 |
| 109 | *4_2_Z_3_ | Duo-Cotecxin | Tablet | Dihydroartemisinin/Piperaquine Phosphate 40/320mg  fixed dose | 110620 | Zhejiang Holley Nanhu  china | 06/2011-  06/2013 |
| 110 | *4_1_Z_3_ | Duo-Cotecxin | Tablet | Dihydroartemisinin/Piperaquine Phosphate 40/320mg  fixed dose | 110123 | Zhejiang Holley Nanhu  China | 01/2011-  01/2013 |
| 111 | 4_5_Z_1_ | Alaxin | Tablet | Dihydroartemisinin/Sulphadoxine BP/Pyrimethamine BP 60/500/25mg | AP-18 | Bliss GVS  India | 08/2011-  07/2014 |
| 112 | 4_6_Z_1_ | Alaxin | Tablet | Dihydroartemisinin/Sulphadoxine BP/Pyrimethamine BP 60/500/25mg | AP-11 | Bliss GVS  India | 01/2010-  01/2013 |
| * Unregistered samples  **Total number of unregistered samples*** **=** 15 | | | | | | | |
