# Supplementary material for: Post-marketing surveillance of anti-malarial medicines used in Malawi
Source: Malar J. 2015 Mar 25;14:127. doi: 10.1186/s12936-015-0637-z (PMC4377194; doi:10.1186/s12936-015-0637-z)
Supplement: Additional file 2: Figure S1. — Map of Malawi showing sampling sites. [file 12936_2015_637_MOESM2_ESM.docx]

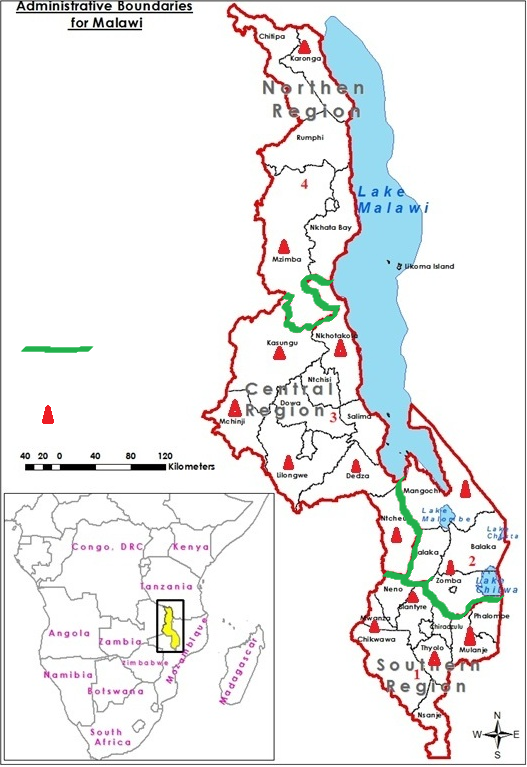


Zonal boundaries

Zonal towns: where sample collections were made.

**Figure 1: Map of Malawi showing sampling sites**

Zonal boundaries

Towns of sample collection within a zone
