# Supplementary material for: Post-marketing surveillance of anti-malarial medicines used in Malawi
Source: Malar J. 2015 Mar 25;14:127. doi: 10.1186/s12936-015-0637-z (PMC4377194; doi:10.1186/s12936-015-0637-z)
Supplement: Additional file 3: — Results of HPLC assay for API content in anti-malarial medicines. [file 12936_2015_637_MOESM3_ESM.docx]

**ADDITIONAL FILE 3**

**RESULTS OF HPLC ASSAY FOR API CONTENT IN ANTI-MALARIAL MEDICINES**

Table 1: Percentage (%) and mass (mg) quantities of results of artesunate API by HPLC methods and their comparison with the manufacturer’s claim and pharmacopoeial requirements. Artesunate tablets must contain at least 90.0% and at most 110.0% of the labelled amount of artesunate on the pack.

| **Code** | **Manufacturer’s Label Claim (mg)** | **HPLC determination of composition of artesunate dosage forms in % and mg quantities (n = 6)** | | **Remarks based on HPLC results** |
| --- | --- | --- | --- | --- |
|  |  | % ± rsd | Quantity (mg) |  |
| *2_4_Y_13_ | **ATS**/S/P:**100**/500/25 | 97.57 ± 0.01 | 98 | C |
| *4Y_13_ | **ATS**/S/P:**100**/500/25 | 94.8 ± 0.1 | 95 | C |
| *3_4_Y_13_ | **ATS**/S/P:**100**/500/25 | 97.65 ± 0.08 | 98 | C |
| *3_2_Y_13_ | **ATS**/S/P:**100**/500/25 | 98.11 ± 0.02 | 98 | C |
| 4_4_Y_12_ | **ATS**/SM/P:**100**/250/12 | 90.4 ± 0.2 | 90 | C |
| 4_2_Y_12_ | **ATS**/SM/P:**200**/500/25 | 92.77 ± 0.02 | 186 | C |
| 4_3_Y_12_ | **ATS**/SM/P:**100**/250/12.5 | 78.22 ± 0.04 | 78 | NC |
| 4_1_Y_12_ | **ATS**/SM/P:**200**/500/25 | 86.9 ± 0.2 | 174 | NC |
| 3_1_Y_12_ | **ATS**/SM/P:**200**/500/25 | 88.96 ± 0.05 | 178 | NC |


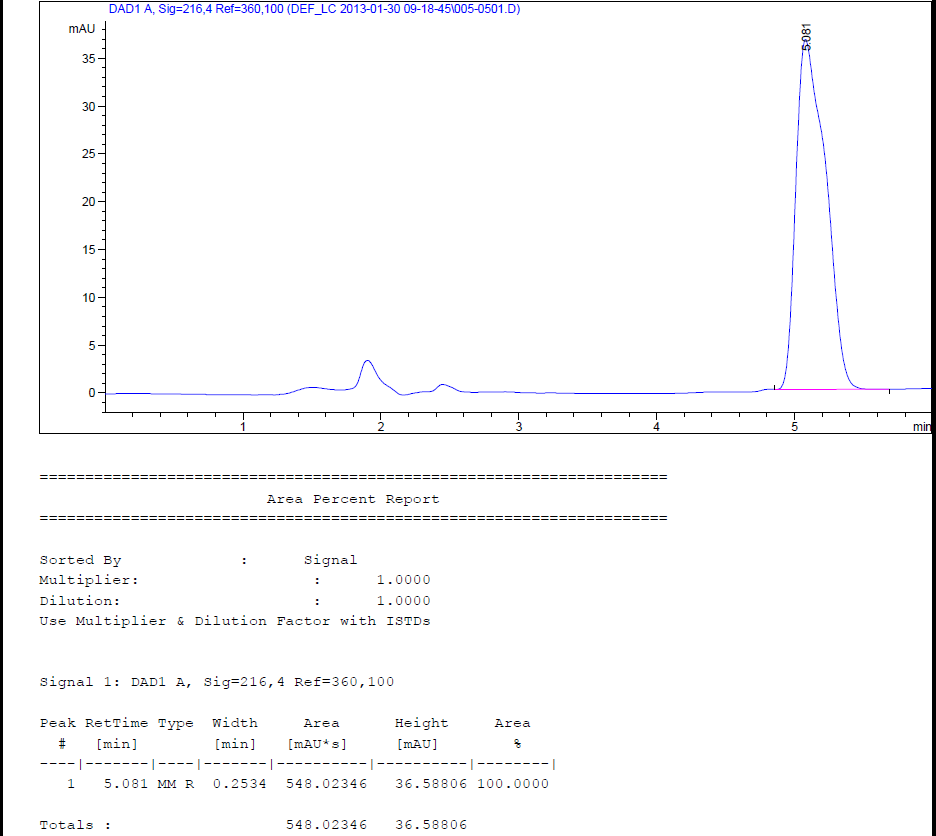


Artesunate peak

Other drug substance

**Chromatogram of a sample solution containing artesunate**

Table 2: Percentage (%) and mass (mg) quantities of results of artemether API by HPLC methods and their comparison with the manufacturer’s claim and pharmacopoeial requirements. Artemether tablets must contain at least 90.0% and at most 110.0% of the labelled amount of artemether on the pack

| **Code** | **Manufacturer’s**  **Label Claim (mg)** | **HPLC determination of composition of artemether dosage forms in % and mg quantities (n = 6)** | | **Remarks based on HPLC results** |
| --- | --- | --- | --- | --- |
|  |  |  |  |  |
|  |  | % ± rsd | Quantity (mg) |  |
| 2X_15_ | **ATM**/LUM:**20**/120 | 144.1 ± 0.7 | 29 | NC overdose |
| 4X_20_ | **ATM**/LUM:**20**/120 | 100 ± 2 | 20 | C |
| 1_1_X_1_ | **ATM**/LUM:**20**/120 | 58.9 ± 0.6 | 12 | NC |
| 1_1_X_11_ | **ATM**/LUM:**180**/1080 | 93 ± 2 | 167 | C |
| 1_1_X_14_ | **ATM**/LUM:**20**/120 | 75.3 ± 0.9 | 15 | NC |
| 1_1_X_17_ | **ATM**/LUM:**80**/480 | 177.8 ± 0.3 | 142 | NC overdose |
| 1_1_X_18_ | **ATM**/LUM:**40**/240 | 26.5 ± 0.8 | 11 | NC |
| 1_1_X_20_ | **ATM**/LUM:**20**/120 | 135 ± 4 | 27 | NC overdose |
| 1_2_X_1_ | **ATM**/LUM:**20**/120 | 119.7 ± 0.8 | 24 | NC overdose |
| 1_2_X_11_ | **ATM**/LUM:**180**/1080 | 103.3 ± 0.8 | 186 | C |
| 1_2_X_14_ | **ATM**/LUM:**20**/120 | 35 ± 2 | 7 | NC |
| 1_3_X_1_ | **ATM**/LUM:**40**/240 | 96.8 ± 0.2 | 39 | C |
| 1_3_X_11_ | **ATM**/LUM:**80**/480 | 39.6 ± 0.5 | 32 | NC |
| 1_4_X_1_ | **ATM**/LUM:**40**/240 | 69 ± 2 | 28 | NC |
| 1_4_X_11_ | **ATM**/LUM:**40**/240 | 82.7 ± 0.3 | 33 | NC |
| 1_5_X_1_ | **ATM**/LUM:**80**/480 | 67.3 ± 0.3 | 54 | NC |
| 1_5_X_11_ | **ATM**/LUM:**20**/120 | 45 ± 1 | 9 | NC |
| 1_6_X_1_ | **ATM**/LUM:**40**/240 | 163.2 ± 0.4 | 65 | NC overdose |
| 1_7_X_1_ | **ATM**/LUM:**80**/480 | 81.205±0 | 65 | NC |
| 1_8_X_1_ | **ATM**/LUM:**20**/120 | 162.7 ± 0.3 | 33 | NC overdose |
| 1_9_X_1_ | **ATM**/LUM:**80**/480 | 103.4 ± 0.4 | 83 | C |
| 2_1_X_1_ | **ATM**/LUM:**20**/120 | 97 ± 1 | 19 | C |
| 2_2_X_1_ | **ATM**/LUM:**80**/480 | 131.5 ± 0.2 | 105 | NC overdose |
| 2_3_X_1_ | **ATM**/LUM:**20**/120 | 149 ± 1 | 30 | NC overdose |
| 2_4_X_14_ | **ATM**/LUM:**20**/120 | 53.2 ± 0.3 | 11 | NC |
| 2_5_X_11_ | **ATM**/LUM:**80**/480 | 113 ± 4 | 90 | NC overdose |
| 3_1_X_1_ | **ATM**/LUM:**20**/120 | 92.4 ± 0.6 | 18 | C |
| 3_2_X_1_ | **ATM**/LUM:**20**/120 | 97 ± 1 | 19 | C |
| 3_1_X_11_ | **ATM**/LUM:**80**/480 | 39 ± 1 | 31 | NC |
| 3_3_X_1_ | **ATM**/LUM:**40**/240 | 81.0 ± 0.3 | 32 | NC |
| 3_4_X_1_ | **ATM**/LUM:**80**/480 | 102.1 ± 0.7 | 82 | C |
| 3_6_X_1_ | **ATM**/LUM:**80**/480 | 104.8 ± 0.3 | 84 | C |
| 4_1_X_11_ | **ATM**/LUM:**20**/120 | 106.2 ± 0.4 | 21 | C |
| 4_2_X_11_ | **ATM**/LUM:**20**/120 | 28 ± 1 | 6 | NC |
| 4_3_X_1_ | **ATM**/LUM:**40**/240 | 173.8 ± 0.3 | 70 | NC overdose |
| 4_4_X_1_ | **ATM**/LUM:**80**/480 | 142.6 ± 0.7 | 114 | NC overdose |
| 4_5_X_12_ | **ATM**/LUM:**180**/1080 | 96.0 ± 0.9 | 173 | C |
| 1_10_X_1_ | **ATM**/LUM:**80**/480 | 124.2 ± 0.5 | 99 | NC overdose |
| 1_6_X_11_ | **ATM**/LUM:**180**/1080 | 99.3 ± 0.9 | 179 | C |
| 1_12_X_1_ | **ATM**/LUM:**80**/480 | 100.9 ± 0 | 81 | C |
| 1_13_X_1_ | **ATM**/LUM:**80**/480 | 140.0 ± 0.5 | 112 | NC overdose |

Table 3: Percentage (%) and mass (mg) quantities of results of lumefantrine API by HPLC methods and their comparison with the manufacturer’s claim and pharmacopoeial requirements. Lumefantrine tablets must contain at least 90.0% and at most 110.0% of the labelled amount of Lumefantrine on the pack

| **Code** | **Manufacturer’s**  **Label Claim (mg)** | **HPLC determination of composition of lumefantrine dosage forms in % and mg quantities**  **(n = 6)** | | **Remarks based on HPLC results** |
| --- | --- | --- | --- | --- |
|  |  |  |  |  |
|  |  | % ± rsd | Quantity (mg) |  |
| 2X_15_ | ATM/**LUM**:20/**120** | 97 ± 2 | 116 | C |
| 4X_20_ | ATM/**LUM**:20/**120** | 119.5 ± 0.4 | 143 | NC overdose |
| 1_1_X_1_ | ATM/**LUM**:20/**120** | 112.5 ± 0.5 | 135 | NC overdose |
| 1_1_X_11_ | ATM/**LUM**:180/**1080** | 127 ± 1 | 1372 | NC overdose |
| 1_1_X_14_ | ATM/**LUM**:20/**120** | 69.3 ± 0.3 | 83 | NC |
| 1_1_X_17_ | ATM/**LUM**:80/**480** | 101.0 ± 0.4 | 485 | C |
| 1_1_X_18_ | ATM/**LUM**:40/**240** | 92.9 ± 0.3 | 223 | C |
| 1_1_X_20_ | ATM/**LUM**:20/**120** | 114.5 ± 0.5 | 137 | NC overdose |
| 1_2_X_1_ | ATM/**LUM**:20/**120** | 113.0 ± 0.4 | 136 | NC |
| 1_2_X_11_ | ATM/**LUM**:180/**1080** | 123 ± 2 | 1328 | NC overdose |
| 1_2_X_14_ | ATM/**LUM**:20/**120** | 95.3 ± 0.2 | 114 | C |
| 1_3_X_1_ | ATM/**LUM**:40/**240** | 81.1 ± 0.3 | 195 | NC |
| 1_3_X_11_ | ATM/**LUM**:80/**480** | 85 ± 1 | 408 | NC |
| 1_4_X_1_ | ATM/**LUM**:40/**240** | 115.0 ± 0.9 | 276 | NC |
| 1_4_X_11_ | ATM/**LUM**:40/**240** | 114.0 ± 0.4 | 274 | NC |
| 1_5_X_1_ | ATM/**LUM**:80/**480** | 110.9 ± 0.2 | 532 | NC overdose |
| 1_5_X_11_ | ATM/**LUM**:20/**120** | 90.5 ± 0.3 | 109 | C |
| 1_6_X_1_ | ATM/**LUM**:40/**240** | 127 ± 1 | 305 | NC overdose |
| 1_7_X_1_ | ATM/**LUM**:80/**480** | 59 ± 1 | 283 | NC |
| 1_8_X_1_ | ATM/**LUM**:20/**120** | 79.19 ± 0.0 | 95 | NC |
| 1_9_X_1_ | ATM/**LUM**:80/**480** | 113.5 ± 0.4 | 545 | NC overdose |
| 2_1_X_1_ | ATM/**LUM**:20/**120** | 113 ± 4 | 136 | NC |
| 2_2_X_1_ | ATM/**LUM**:80/**480** | 63 ± 2 | 302 | NC |
| 2_3_X_1_ | ATM/**LUM**:20/**120** | 103.3 ± 0.3 | 124 | C |
| 2_4_X_14_ | ATM/**LUM**:20/**120** | 117.5 ± 0.8 | 141 | NC overdose |
| 2_5_X_11_ | ATM/**LUM**:80/**480** | 112.2 ± 0.6 | 539 | NC |
| 3_1_X_1_ | ATM/**LUM**:20/**120** | 116.9 ± 0.5 | 140 | NC overdose |
| 3_2_X_1_ | ATM/**LUM**:20/**120** | 117 ± 2 | 140 | NC overdose |
| 3_1_X_11_ | ATM/**LUM**:80/**480** | 118.6 ± 0.6 | 569 | NC overdose |
| 3_3_X_1_ | ATM/**LUM**:40/**240** | 108.0 ± 0.2 | 259 | C |
| 3_4_X_1_ | ATM/**LUM**:80/**480** | 111.6 ± 0.3 | 536 | NC overdose |
| 3_6_X_1_ | ATM/**LUM**:80/**480** | 103.3 ± 0.1 | 496 | C |
| 4_1_X_11_ | ATM/**LUM**:20/**120** | 50 ± 6 | 60 | NC |
| 4_2_X_11_ | ATM/**LUM**:20/**120** | 88 ± 2 | 106 | NC |
| 4_3_X_1_ | ATM/**LUM**:40/**240** | 81.8 ± 0.3 | 196 | NC |
| 4_4_X_1_ | ATM/**LUM**:80/**480** | 104.0 ± 0.2 | 499 | C |
| 4_5_X_12_ | ATM/**LUM**:180/**1080** | 129 ± 2 | 1393 | NC overdose |
| 1_10_X_1_ | ATM/**LUM**:80/**480** | 63 ± 1 | 302 | NC |
| 1_6_X_11_ | ATM/**LUM**:180/**1080** | 119.3 ± 0.1 | 1288 | NC overdose |
| 1_12_X_1_ | ATM/**LUM**:80/**480** | 102.5 ± 0 | 492 | C |
| 1_13_X_1_ | ATM/**LUM**:80/**480** | 109.96±0.0 | 528 | C |

**`**

Table 4: Percentage (%) and mass (mg) quantities of results of dihydroartemisinin (Artenimol) API by HPLC methods and their comparisons with the manufacturer’s claim and pharmacopoeial requirements. Dihydroartemisinin tablets must contain at least 90.0% and at most 110.0% of the labelled amount of Dihydroartemisinin on the pack

| **Code** | **Manufacturer’s**  **Label Claim (mg)** | **HPLC determination of composition of dihydroartemisinin dosage forms in % and mg quantities**  **(n = 6)** | | **Remarks based on HPLC results** |
| --- | --- | --- | --- | --- |
|  |  |  |  |  |
|  |  | % ± rsd | Quantity (mg) |  |
| 1_1_Z_3_ | **DHA**/PP:**40**/320 | 98.7 ± 0.2 | 39 | C |
| 1_2_Z_3_ | **DHA**/PP:**40**/320 | 86 ± 2 | 34 | NC |
| 1_4_Z_3_ | **DHA**/PP:**40**/320 | 70 ± 1 | 28 | NC |
| 1_6_Z_1_ | **DHA**/PP:**40**/320 | 97.5 ± 0.9 | 39 | C |
| 1_7_Z_1_ | **DHA**/PP:**40**/320 | 85 ± 1 | 34 | NC |
| 2_6_Z_1_ | **DHA**/PP:**40**/320 | 103 ± 1 | 41 | C |
| 2_7_Z_1_ | **DHA**/PP:**40**/320 | 88 ± 5 | 35 | NC |
| 3_2_Z_3_ | **DHA**/PP:**40**/320 | 73 ± 2 | 29 | NC |
| 3_3_Z_3_ | **DHA**/PP:**40**/320 | 71 ± 1 | 28 | NC |
| 3_7_Z_1_ | **DHA**/PP:**40**/320 | 101 ± 6 | 40 | C |
| 4_1_Z_3_ | **DHA**/PP:**40**/320 | 74 ± 3 | 30 | NC |
| 4_2_Z_3_ | **DHA**/PP:**40**/320 | 87 ± 1 | 35 | NC |
| 4_3_Z_3_ | **DHA**/PP:**40**/320 | 71 ± 3 | 28 | NC |
| 4_4_Z_3_ | **DHA**/PP:**40**/320 | 88 ± 1 | 35 | NC |
| 1_5_Z_1_ | **DHA**/S/P:**60**/500/25 | 51.4 ± 0.2 | 31 | NC |
| 1_3_Z_1_ | **DHA**/S/P:**60**/500/25 | 56.8 ± 0.2 | 34 | NC |
| 1_2_Z_1_ | **DHA**/S/P:**60**/500/25 | 52.5 ± 0.1 | 32 | NC |
| 1_4_Z_1_ | **DHA**/S/P:**60**/500/25 | 61.6 ± 0.1 | 37 | NC |
| 2_1_Z_1_ | **DHA**/S/P:**60**/500/25 | 56.48±0.09 | 34 | NC |
| 2_3_Z_1_ | **DHA**/S/P:**60**/500/25 | 54.4 ± 0.2 | 33 | N C |
| 2_4_Z_1_ | **DHA**/S/P:**60**/500/25 | 52.5 ± 0.1 | 32 | NC |
| 2_5_Z_1_ | **DHA**/S/P:**60**/500/25 | 51.25±0.06 | 31 | NC |
| 3_4_Z_1_ | **DHA**/S/P:**60**/500/25 | 51.4 ± 0.1 | 31 | NC |
| 3_5_Z_1_ | **DHA**/S/P:**60**/500/25 | 51.8 ± 0.1 | 31 | NC |
| 4_5_Z_1_ | **DHA**/S/P:**60**/500/25 | 51.67±0.05 | 31 | NC |
| 4_6_Z_1_ | **DHA**/S/P:**60**/500/25 | 51.92±0.09 | 31 | NC |

Piperaquine peak

Dihydroartemisinin peak

Related substance

Chromatogram of a sample solution containing dihydroartemisinin

Table 5: Percentage (%) and Mass (mg) quantities of results of sulphadoxine API by HPLC methods and their comparison with the manufacturer’s claim and pharmacopoeial requirements. Sulphadoxine tablets must contain at least 90.0% and at most 110.0% of the labelled amount of Sulphadoxine on the pack

| **Code** | **Manufacturer’s**  **Label Claim (mg)** | **HPLC determination of composition of sulphadoxine dosage forms in % and mg quantities (n = 6)** | | **Remarks based on HPLC results** |
| --- | --- | --- | --- | --- |
|  |  |  |  |  |
|  |  | % ± rsd | Quantity (mg) |  |
| 1_1_P_10_ | **S/P**:**500**/25 | 78 ± 2 | 390 | NC |
| 1_1_P_2_ | **S/P**:**500**/25 | 87 ± 2 | 435 | NC |
| 1_2_P_2_ | **S/P**:**500**/25 | 67 ± 4 | 335 | NC |
| 1_4_P_10_ | **S/P**:**500**/25 | 85 ± 2 | 425 | NC |
| 1_4_P_2_ | **S/P**:**500**/25 | 82 ± 2 | 410 | NC |
| 1_6_P_10_ | **S/P**:**500**/25 | 83 ± 3 | 415 | NC |
| 2_1_P_15_ | **S/P**:**500**/25 | 82 ± 3 | 410 | NC |
| 2_1_P_2_ | **S/P**:**500**/25 | 77 ± 1 | 385 | NC |
| 2_2_P_2_ | **S/P**:**500**/25 | 83 ± 2 | 415 | NC |
| 2_3_P_2_ | **S/P**:**500**/25 | 96 ± 2 | 480 | C |
| 3_1_P_10_ | **S/P**:**500**/25 | 83 ± 2 | 415 | NC |
| 3_1_P_2_ | **S/P**:**500**/25 | 97 ± 3 | 485 | C |
| 3_2_P_10_ | **S/P**:**500**/25 | 81 ± 2 | 405 | NC |
| 3_2_P_2_ | **S/P**:**500**/25 | 85 ± 2 | 425 | NC |
| 3_3_P_10_ | **S/P**:**500**/25 | 82 ± 2 | 410 | NC |
| 3_5_P_2_ | **S/P**:**500**/25 | 53 ± 3 | 265 | NC |
| 3_7_P_15_ | **S/P**:**500**/25 | 71 ± 2 | 355 | NC |
| 3_8_P_15_ | **S/P**:**500**/25 | 91.7 ± 0.2 | 459 | C |
| 4_1_P_2_ | **S/P**:**500**/25 | 89 ± 3 | 445 | NC |
| 4_2_P_2_ | **S/P**:**500**/25 | 47 ± 2 | 235 | NC |
| 4_4_P_2_ | **S/P**:**500**/25 | 85.17 ± 0.06 | 426 | NC |
| 4_5_P_2_ | **S/P**:**500**/25 | 85.1 ± 0.2 | 426 | NC |
| 4_8_P_5_ | **S/P**:**500**/25 | 85 ± 2 | 425 | NC |
| *2_4_Y_13_ | ATS/**S/P**:100/**500**/25 | 72 ± 2 | 360 | NC |
| *4Y_13_ | ATS/**S/P**:100/**500**/25 | 89 ± 2 | 445 | NC |
| *3_4_Y_13_ | ATS/**S/P**:100/**500**/25 | 74 ± 4 | 370 | NC |
| *3_2_Y_13_ | ATS/**S/P**:100/**500**/25 | 68 ± 5 | 340 | NC |
| 1_5_Z_1_ | DHA/**S/P**:60/**500**/25 | 85.694 ± 0 | 428 | NC |
| 1_3_Z_1_ | DHA/**S/P**:60/**500**/25 | 86 ± 2 | 430 | NC |
| 1_2_Z_1_ | DHA/**S/P**:60/**500**/25 | 82.7 ± 0.9 | 414 | NC |
| 1_4_Z_1_ | DHA/**S/P**:60/**500**/25 | 79 ± 2 | 395 | NC |
| 2_1_Z_1_ | DHA/**S/P**:60/**500**/25 | 77 ± 2 | 385 | NC |
| 2_3_Z_1_ | DHA/**S/P**:60/**500**/25 | 90 ± 2 | 450 | C |
| 2_4_Z_1_ | DHA/**S/P**:60/**500**/25 | 80 ± 2 | 400 | NC |
| 2_5_Z_1_ | DHA/**S/P**:60/**500**/25 | 86.7 ± 0.6 | 434 | NC |
| 3_4_Z_1_ | DHA/**S/P**:60/**500**/25 | 85.4 ± 0.2 | 427 | NC |
| 3_5_Z_1_ | DHA/**S/P**:60/**500**/25 | 84.9 ± 0.1 | 425 | NC |
| 4_5_Z_1_ | DHA/**S/P**:60/**500**/25 | 87.5 ± 0.8 | 438 | NC |
| 4_6_Z_1_ | DHA/**S/P**:60/**500**/25 | 75.2 ± 0.1 | 376 | NC |
| 4_4_Y_12_ | ATS/**SM/P**:100/**250**/12.5 | 90 ± 3 | 225 | C |
| 4_2_Y_12_ | ATS/**SM/P**:200/**500**/25 | 88.6 ± 0.2 | 443 | NC |
| 4_3_Y_12_ | ATS/**SM/P**:100/**250**/12.5 | 87 ± 2 | 218 | NC |
| 4_1_Y_12_ | ATS/**SM/P**:200/**500**/25 | 87.9 ± 0.5 | 440 | NC |
| 3_1_Y_12_ | ATS/**SM/P**:200/**500**/25 | 86.6 ± 0.2 | 217 | NC |

Pyrimethamine peak

Sulphadoxine peak

**Chromatogram of a sample containing sulphadoxine and pyrimethamine**

Table 6: Percentage (%) and Mass (mg) quantities of results of pyrimethamine API by HPLC methods and their comparisons with the manufacturer’s claim and pharmacopoeial requirements. Pyrimethamine tablets must contain at least 90.0% and at most 110.0% of the labelled amount of Pyrimethamine on the pack

| **Code** | **Manufacturer’s**  **Label Claim (mg)** | **HPLC determination of composition of pyrimethamine dosage forms in % and mg quantities**  **(n = 6)** | | **Remarks based on HPLC results** |
| --- | --- | --- | --- | --- |
|  |  |  |  |  |
|  |  | % ± rsd | Quantity (mg) |  |
| 1_1_P_10_ | S/P:500/**25** | 92±0 | 23 | C |
| 1_1_P_2_ | S/P:500/**25** | 114±0 | 29 | NC overdose |
| 1_2_P_2_ | S/P:500/**25** | 76±0 | 19 | NC |
| 1_4_P_10_ | S/P:500/**25** | 110±0 | 28 | C |
| 1_4_P_2_ | S/P:500/**25** | 93±0 | 23 | C |
| 1_6_P_10_ | S/P:500/**25** | 92±0 | 23 | C |
| 2_1_P_15_ | S/P:500/**25** | 93±0 | 23 | C |
| 2_1_P_2_ | S/P:500/**25** | 89±0 | 22 | NC |
| 2_2_P_2_ | S/P:500/**25** | 64±0 | 16 | NC |
| 2_3_P_2_ | S/P:500/**25** | 122±0 | 31 | NC overdose |
| 3_1_P_10_ | S/P:500/**25** | 83±0 | 21 | NC |
| 3_1_P_2_ | S/P:500/**25** | 97±0 | 24 | C |
| 3_2_P_10_ | S/P:500/**25** | 94±0 | 24 | C |
| 3_2_P_2_ | S/P:500/**25** | 96±0 | 24 | C |
| 3_3_P_10_ | S/P:500/**25** | 87±0 | 22 | NC |
| 3_5_P_2_ | S/P:500/**25** | 48±0 | 12 | NC |
| 3_7_P_15_ | S/P:500/**25** | 78±0 | 20 | NC |
| 3_8_P_15_ | S/P:500/**25** | 103±0 | 26 | C |
| 4_1_P_2_ | S/P:500/**25** | 113±0 | 28 | NC overdose |
| 4_2_P_2_ | S/P:500/**25** | 51±0 | 13 | NC |
| 4_4_P_2_ | S/P:500/**25** | 102±0 | 26 | C |
| 4_5_P_2_ | S/P:500/**25** | 108±0 | 27 | C |
| 4_8_P_5_ | S/P:500/**25** | 110±0 | 28 | C |
| *2_4_Y_13_ | ATS/S/P:100/500/**25** | 91±0 | 23 | C |
| *4Y_13_ | ATS/S/P:100/500/**25** | 98±0 | 25 | C |
| *3_4_Y_13_ | ATS/S/P:100/500/**25** | 70±0 | 18 | NC |
| *3_2_Y_13_ | ATS/S/P:100/500/**25** | 78±0 | 20 | NC |
| 1_5_Z_1_ | DHA/S/P:60/500/**25** | 94±0 | 24 | C |
| 1_3_Z_1_ | DHA/S/P:60/500/**25** | 91±0 | 23 | C |
| 1_2_Z_1_ | DHA/S/P:60/500/**25** | 98±0 | 25 | C |
| 1_4_Z_1_ | DHA/S/P:60/500/**25** | 106±0 | 27 | C |
| 2_1_Z_1_ | DHA/S/P:60/500/**25** | 105±0 | 26 | C |
| 2_3_Z_1_ | DHA/S/P:60/500/**25** | 100±0 | 25 | C |
| 2_4_Z_1_ | DHA/S/P:60/500/**25** | 95±0 | 24 | C |
| 2_5_Z_1_ | DHA/S/P:60/500/**25** | 90±0 | 23 | C |
| 3_4_Z_1_ | DHA/S/P:60/500/**25** | 98±0 | 25 | C |
| 3_5_Z_1_ | DHA/S/P:60/500/**25** | 98±0 | 25 | C |
| 4_5_Z_1_ | DHA/S/P:60/500/**25** | 103±0. | 26 | C |
| 4_6_Z_1_ | DHA/S/P:60/500/**25** | 99±0 | 25 | C |
| 4_4_Y_12_ | ATS/SM/P:100/250/**12.5** | 119±0 | 15 | NC overdose |
| 4_2_Y_12_ | ATS/SM/P:200/500/**25** | 116±0 | 29 | NC overdose |
| 4_3_Y_12_ | ATS/SM/P:100/250/**12.5** | 113±0 | 14 | NC overdose |
| 4_1_Y_12_ | ATS/SM/P:200/500/**25** | 101.73±0 | 25 | C |
| 3_1_Y_12_ | ATS/SM/P:200/500/**25** | 110±0 | 14 | C |

Table 7: Percentage (%) and Mass (mg) quantities of results of quinine API by HPLC methods and their comparisons with the manufacturer’s claim and pharmacopoeial requirements. Quinine tablets must contain at least 90.0% and at most 110.0% of the labelled amount of Quinine on the pack

| **Code** | **Manufacturer’s**  **Label Claim (mg)** | **HPLC determination of composition of quinine dosage forms in % and mg quantities**  **(n = 6)** | | **Remarks based on HPLC results** |
| --- | --- | --- | --- | --- |
|  |  |  |  |  |
|  |  | %  ±rsd | Quantity  (mg) |  |
| 1_1_V_5_ | **QN:50mg/5ml** | 109.7 ± 0.3 | 55 | C |
| 1_2_V_5_ | **QN:50mg/5ml** | 120 ± 1 | 60 | NC overdose |
| 1_3_V_5_ | **QN:50mg/5ml** | 112 ± 1 | 56 | NC overdose |
| 4_1_Q_6_ | **QN:150mg/5ml** | 102 ± 4 | 153 | C |
| 4_2_Q_6_ | **QN:150mg/5ml** | 56 ± 7 | 84 | NC |
| 4_3_Q_6_ | **QN:150mg/5ml** | 97 ± 4 | 146 | C |
| 4V_5_ | **QN:50mg/5ml** | 112 ± 2 | 56 | NC |
| 4_1_R_8_ | **QN:300mg/ml** | 291 ± 1 | 873 | NC overdose |
| 4_2_R_4_ | **QN:100mg/5ml** | 151 ± 1 | 151 | NC overdose |
| 4_3_R_4_ | **QN:100mg/5ml** | 156 ± 2 | 156 | NC overdose |
| 3_1_Q_6_ | **QN:150mg/5ml** | 110.4 ± 0.1 | 166 | C |
| 3_2_Q_6_ | **QN:150mg/5ml** | 110.5 ± 0.1 | 166 | C |
| 3_3_Q_6_ | **QN:150mg/5ml** | 122.1 ± 0.8 | 183 | NC overdose |

Related substances

Quinine peak

Chromatogram of a sample solution containing quinine
